# Spotlight influenza: Laboratory-confirmed seasonal influenza in people with acute respiratory illness: a literature review and meta-analysis, WHO European Region, 2004 to 2017

**DOI:** 10.2807/1560-7917.ES.2021.26.39.2000343

**Published:** 2021-09-30

**Authors:** Sara Belazi, Sonja J Olsen, Caroline Brown, Helen K Green, Piers Mook, Jonathan Nguyen-Van-Tam, Pasi Penttinen, Louise Lansbury

**Affiliations:** 1 Division of Epidemiology and Public Health, University of Nottingham, Nottingham, United Kingdom; 2 WHO Regional Office for Europe, Copenhagen, Denmark; 3 Public Health England, London, United Kingdom; 4 European Centre for Disease Prevention and Control (ECDC), Solna, Sweden

**Keywords:** influenza human, influenza A virus, influenza B virus, acute respiratory infection, influenza-like illness, medically-attended

## Abstract

**Background:**

Across the World Health Organization European Region, there are few estimates of the proportion of people seeking medical care for influenza-like illness or acute respiratory infections and who have laboratory-confirmed seasonal influenza infection.

**Methods:**

We conducted a meta-analysis of data extracted from studies published between 2004 and 2017 and from sentinel data from the European surveillance system (TESSy) between 2004 and 2018. We pooled within-season estimates by influenza type/subtype, setting (outpatient (OP)/inpatient (IP)) and age group to estimate the proportion of people tested who have laboratory-confirmed and medically-attended seasonal influenza in Europe.

**Results:**

In the literature review, the pooled proportion for all influenza types was 33% (95% confidence interval (CI): 30–36), higher among OP 36% (95% CI: 33–40) than IP 24% (95% CI: 20–29). Pooled estimates for all influenza types by age group were: 0–17 years, 26% (22–31); 18–64 years, 41% (32–50); ≥ 65 years, 33% (27–40). From TESSy data, 33% (31–34) of OP and 24% (21–27) of IP were positive. The highest proportion of influenza A was in people aged 18–64 years (22%, 16–29). By subtype, A(H1N1)pdm09 was highest in 18–64 year-olds (16%, 11–21%) whereas A(H3N2) was highest in those ≥ 65 years (10%, 2–22). For influenza B, the highest proportion of infections was in those aged 18–64 years (15%, 9–24).

**Conclusions:**

Laboratory-confirmed influenza accounted for approximately one third of all acute respiratory infections for which medical care was sought during the influenza season.

## Introduction

Seasonal and pandemic influenza are global public health problems associated with substantial clinical burden [1]. However, seasonal influenza causes higher cumulative morbidity and mortality as it affects populations on an annual basis [1,2]. The epidemiological impact of influenza varies from season to season, between geographic areas and according to the circulating antigenic variants of the main influenza types, A and B [2-6]. Within a population, the clinical impact in at-risk subgroups for the development of serious influenza-related complications (children, elderly people, pregnant women and people with underlying co-morbidities) is greater than in the rest of the population [2].

Influenza infection is common across the World Health Organization (WHO) European Region. Asymptomatic infections account for 16% to 85% of seasonal influenza infections, depending on study design and testing method [1,7]. Most symptomatic individuals experience mild and self-limiting illness [2,3]. Accurate diagnosis of influenza A or B in people seeking medical care solely on the basis of clinical criteria is difficult because the signs and symptoms of influenza overlap with those of many other respiratory viral pathogens which co-circulate with influenza every winter in temperate regions.

Laboratory-testing identifies the specific causative virus, but in clinical practice few patients presenting with signs and symptoms suggestive of influenza are actually tested [8]. Therefore, the actual contribution of influenza viruses to total respiratory illness remains uncertain, especially in primary care settings where laboratory testing is rarely undertaken.

Since the early 2000s, large investments by the United States (US) and other countries have supported development of global capacity for influenza surveillance. Many networks for influenza surveillance now exist globally and in the European Region specifically [9-12], where the WHO Regional Office for Europe (WHO/Europe) and European Centre for Disease Prevention and Control (ECDC) jointly coordinate the collection and analysis of surveillance data provided by the countries (European Surveillance System (TESSy).

In 2015, we conducted a scoping literature review on the burden of influenza within the WHO European Region (unpublished data). This provided an overview of the general burden caused by seasonal influenza and highlighted the lack of data from eastern European countries. However, some of the estimates provided were derived from symptom-based endpoints (e.g. influenza-like illness (ILI) or severe acute respiratory infection (SARI), which made it impossible to compare the clinical burden of laboratory-confirmed influenza across countries.

In this study we aim to estimate the proportion of laboratory-confirmed influenza in the WHO European Region among people seeking medical care who were clinically diagnosed with acute respiratory infection (ARI) or ILI and were tested for respiratory viruses, including influenza. Two methods are presented: a literature review and meta-analysis of data published in the literature from 2004 to 2017 and a meta-analysis of seasonal influenza data from the European Surveillance System (TESSy) between 2004 and 2018.

## Methods

### Literature review

The study is reported according to Preferred Reporting Items for Systematic Reviews and Meta-Analyses (PRISMA) guidelines [13]. We identified articles which reported quantitative data on laboratory-confirmed influenza infections in people seeking medical attention for ILI/ARI in the WHO European Region.

We searched Medline on 19 September 2017 using a search strategy devised by one author (LL, Supplement S1). The strategy sought studies of any design, in any language performed between 2015 and 2017 (influenza seasons up until 2016/17) which were conducted in countries within the WHO European Region [14]. Studies were included if they reported on within-season influenza positivity data for at least one full influenza season (from October to May of the following year) on symptomatic and medically-attended acute respiratory illness in patients of any age, and in whom influenza virus infection was confirmed by culture or reverse transcription PCR (RT-PCR).

Additionally, we searched the reference lists of included studies, relevant systematic reviews [15-19] and the references included in an unpublished scoping review we conducted previously that looked at studies conducted between 2004–2015. We included study populations of all ages or those in stratified age groups, and across all healthcare settings including primary care/ambulatory outpatients (OP) and hospitalised in-patients (IP).

Studies were excluded if the total number of specimens tested each year was fewer than 50, or if they reported on outbreaks in closed or semi-closed communities or institutions (e.g., nursing homes, army bases, or religious groups) where results would not be representative of the wider population. Studies were also excluded if data presented were combined for more than one season or more than one pathogen with no influenza data reported separately. We also excluded studies without a clear sampling strategy or where participants were sampled at the discretion of the treating clinician, which could introduce bias.

One of the authors (SB) screened all titles and abstracts, then the same author conducted a full-text review of eligible papers and extracted the following variables: influenza season, country, age group, laboratory testing method, healthcare setting, case definition, total number of symptomatic subjects tested and number of subjects positive for influenza. If data for separate influenza types and subtypes were presented, the number of positive subjects for each type and subtype were also extracted. The percentage of positive subjects was calculated using the overall number of subjects tested as a denominator, and the number of positive subjects as a numerator (aggregated influenza, types and subtypes according to the data presented in each study).

For assessment of study quality, one of the authors (SB) used a modified version of the Newcastle-Ottawa assessment scale for cohort studies [20]. Indicators used to assess quality were the following: representativeness (geographic, age and general representativeness) of the subjects tested, assessment of the outcome (sensitivity of symptoms prompting laboratory testing such as number of symptoms and having a clear case definition), and laboratory method. Some indicators were not applicable and therefore excluded (i.e., representativeness of exposed cohort, ascertainment of exposure and demonstration that outcome was not present at the start of the study). Other indicators (comparability of cohorts and adequacy and length of follow up) were also excluded since we only included studies that reported on at least one complete season of data.

### The European Surveillance System (TESSy) data

We analysed laboratory-confirmed influenza detection data reported to the European Surveillance System (TESSy) which is hosted by the European Centre for Disease Prevention and Control (ECDC) as a part of the surveillance of influenza in the WHO European Region and jointly coordinated with WHO Europe. Sentinel influenza surveillance is conducted in a representative subset of sites and coordinated by national or sub-national networks, with systematic sampling of patients who meet pre-defined case definitions. Data were provided by Albania, Armenia, Austria, Azerbaijan, Belarus, Belgium, Bulgaria, Croatia, Czech Republic, Denmark, Estonia, Finland, France, Georgia, Germany, Greece, Hungary, Ireland, Israel, Italy, Latvia, Lithuania, Luxembourg, North Macedonia, Malta, Republic of Moldova, the Netherlands, Norway, Poland, Portugal, Kazakhstan, Kyrgyzstan, Portugal, Romania, Russia, Serbia, Slovakia, Slovenia, Spain, Sweden, Switzerland, Tajikistan, Turkey, United Kingdom (UK), Ukraine and Uzbekistan. Data on the duration of participation of these countries are listed in Supplement S2.

Data submitted by week and country for the period 2004 to 2018 (weeks 40 to 20) were extracted from TESSy on 29 August 2018. Separately, the total number of specimens collected from patients presenting at sentinel primary care sites who met the case definitions for ILI or ARI, and specimens from hospitalised patients meeting the case definition for SARI, were calculated by country and influenza season from 2004/05 up to the 2017/18 season. The corresponding total number of detections by influenza virus type and subtype (for influenza A) was also calculated. Country-seasons were excluded if there were fewer than 50 specimens or less than 20 weeks of data submitted to TESSy. The proportion of sampled patients that tested positive for any influenza virus, influenza A virus and influenza B virus were calculated by country-season.

### Data analysis

We extracted data from the literature review and those derived from the TESSy dataset to a Microsoft Office Excel 2013 spreadsheet. As we anticipated a degree of heterogeneity due to the observational nature of the included studies, we used a generic variance approach based on a random effects model (DerSimonian-Laird weights method) [21] to estimate the pooled proportion of laboratory-confirmed influenza virus identified in tested patients, stabilising the variances using the Freeman-Tukey double arcsine transformation so that studies with proportions close to 0% or 100% were appropriately estimated [22]. Exact binomial confidence intervals were computed for outcomes. The main outcome was the proportion (and 95% confidence interval (CI)) of laboratory-confirmed influenza in people with ARI or ILI symptoms who sought medical care and were tested for influenza (the denominator). Data from the literature review and TESSy were analysed separately.

When analysing TESSy data, people with ARI/ILI were classified as OP since the data were derived from surveillance of mild respiratory disease due to influenza at primary care level, whereas those with SARI were classified as hospitalised since these data originated from sentinel surveillance of hospitalised cases presenting with severe disease [23]. The denominator for the latter was the number of people with SARI who were tested for influenza. Heterogeneity between the studies was assessed using the I^2^ statistic.

Initially, an analysis of all types of influenza (aggregated influenza) and for all ages was carried out. To investigate potential sources of heterogeneity, we performed subgroup analyses by influenza virus type (influenza A and B) and subtype (A(H1N1), A(H3N2)), age group, healthcare setting (OP vs IP), and whether the inclusion of fever in the case definition had an impact. To further investigate the high heterogeneity, we undertook an I^2^ sensitivity analysis by excluding datasets in which the estimated proportion was furthest from the overall estimated pooled proportion. We excluded data collected during the 2009 pandemic: influenza A(H1N1) was stratified into pre-pandemic seasons (A(H1N1), up to and including the 2008/09 season) and post-pandemic seasons (A(H1N1)pdm09, 2010/11 onwards) and analysed separately because population susceptibility to these two viruses will have been markedly different. Age groups in included studies were not uniform, so for the purpose of this analysis we created the following categories to best fit the majority of the data: 0–17 years, 18–64 years and ≥ 65 years. All analyses were conducted using the metaprop command in Stata.

### Ethical statement

Ethical approval was not required since the review of the literature is based on published secondary data. ECDC has a legal basis to collect surveillance data (Decision number: 1082/2013/EU) [24] and no ethical approval is needed for data analysis.

## Results

### Literature review

**Figure 1:**
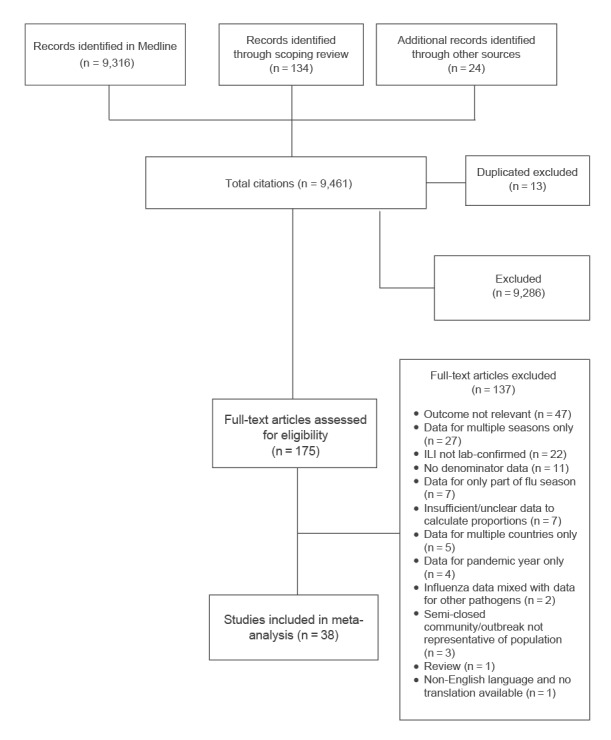
PRISMA flowchart displaying number of articles identified and screened at each stage of the literature review (n =  9,461)

We searched the Medline database and identified 9,316 eligible manuscripts. In total we screened 9,461 references by title and abstract. Of these, we assessed 175 full text articles and 38 met the inclusion criteria (Figure 1 and Supplement S3) [11,12,25-60].

ILI: influenza-like illness.

#### Study characteristics

Studies reported data from 25 European countries (Albania, Armenia, Austria, Belarus, Bulgaria, Czechia, Finland, France, Georgia, Germany, Greece, Italy, Kazakhstan, Kyrgyzstan, the Netherlands, Portugal, Romania, Russia, Slovenia, Spain, Sweden, Switzerland, Turkey, UK and Ukraine). Of these, six studies were conducted in Italy and six in the UK. One study was conducted in each of the following countries: Albania, Armenia, Georgia, Kazakhstan, Romania, Belarus, Ukraine and Kyrgyzstan. Five studies reported data from more than one country.

The studies included covered influenza seasons 1996/97 to 2014/15. Of the 38 included studies, all study designs were cross-sectional except for two test negative case–control studies. Detection of influenza viruses was achieved by RT-PCR with or without culture in 32 studies and by culture alone in six studies [46,52,54,55,57,58]. A sensitivity analysis was conducted excluding the culture-only studies (Supplement S4); however, this had a negligible effect on the pooled estimates. Ten studies were conducted among IPs while the remainder were among OPs.

#### Risk of bias assessment

Among the 38 included studies, quality was determined to be high or intermediate in 24 for geographical representativeness, in 29 for age representativeness, in 38 for general representativeness, in 31 for sensitivity of symptoms, and in 38 for laboratory methods (Supplement S5 and S6). Age representation was rated as low in 10 of the 38 studies because data were combined for all ages. Sensitivity of symptoms was rated low in seven studies that required two or more specific symptoms for inclusion of subjects.

#### Meta-analysis

**Figure 2:**
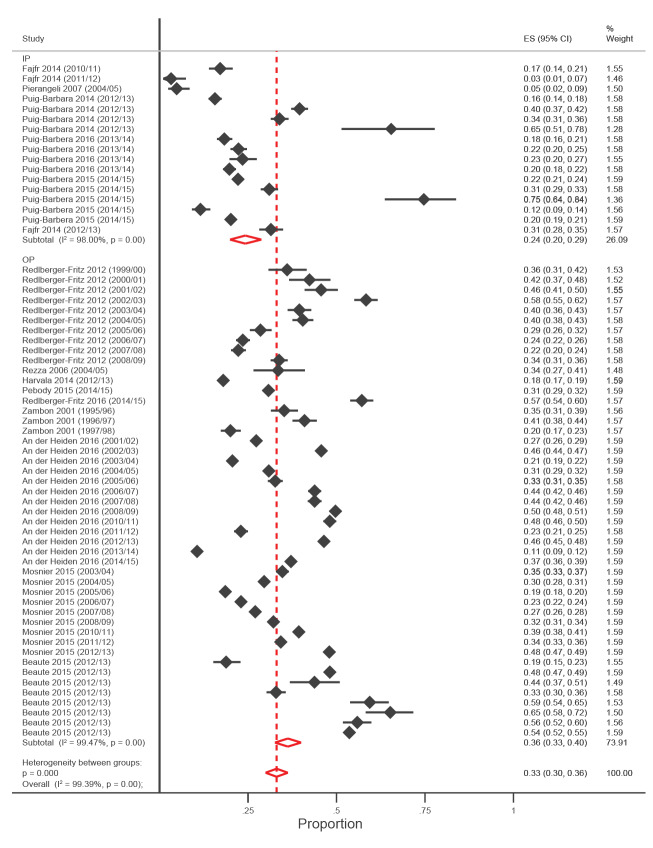
Forest plot of studies from literature review showing proportion of all influenza viruses grouped by healthcare setting, WHO Europe Region, 2004–2017 (n = 145,057)

CI: confidence interval; ES: effect size; IP: inpatient; OP: outpatient; WHO: World Health Organization.

The pooled estimates of the proportion of people of any age who were tested and who were positive for any type of influenza were 36% (95% CI: 33–40, I^2^ = 99.5%, nine studies, 47 datasets) for OPs, and 24% (95% CI: 20–29, I^2^ = 98.4%, five studies, 16 datasets) for IPs (Figure 2). Proportions by influenza type and subtype are presented in Table 1. The I^2^ sensitivity analyses in which 10 datasets where the proportion of positivity was <5% or >50% were excluded slightly reduced the estimates but did not significantly decrease the observed heterogeneity of 33% (95% CI: 30–37, I^2^ = 99.3%) and 23% (95% CI: 19–27, I^2^ = 97.8%) for OPs and IPs respectively.

Several studies reported findings for more than one season and more than one country.

**Table 1:** Pooled estimates of the proportion of medically-attended people with ARI/ILI testing positive for influenza viruses, overall and by age group from meta-analysis, WHO European Region, 2004 to 2017

Influenza virus	Proportion positive %	95% CI	Ref		Proportion of medically-attended people with ARI/ILI testing positive for influenza viruses								
					0–17 years			18–64 years			≥ 65 years		
					%	95% Cl	Number of studies	%	95% Cl	Number of studies	%	95% Cl	Number of studies
Any influenza virus													
Overall	33	30–36	[11,25-34,36,37,59]		26	22–31	21	41	32–50	4	33	27–40	5
Outpatient	36	33–40	[11,28-31,34,36,37,59]		25	20–30	18	41	32–50	4	33	27–40	5
Inpatient	24	20–29	[25-27,32,33]		9	6–12	3	Na	Na	Na	Na	Na	Na
Influenza A													
Overall	24	21–26	[11,25-32,36,37]		12	9–15	12	22	16–29	4	18	8–31	4
Outpatient	24	21–28	[11,28-31,36,37]		14	11–18	9	22	16–29	4	18	8–31	4
Inpatient	20	16–26	[25-27,32]		6	4–9	3	Na	Na	Na	Na	Na	Na
Influenza B													
Overall	7	6–10	[11,12,25-34,36-38,48,60]		6	3–8	17	15	9–24	4	10	3–19	5
Outpatient	9	6–12	[11,28-31,34,36,37,48,60]		9	5–14	13	15	9–24	4	10	3–19	5
Inpatient	5	3–8	[12,25-27,32,33,38]		3	1–5	4	Na	Na	Na	Na	Na	Na
Influenza A(H1N1)^a^													
Outpatient	3	1–5		[28,36]	3	1–6	4	Na	Na	Na	Na	Na	Na
Inpatient	Na	Na		Na	Na	Na	Na	Na	Na	Na	Na	Na	Na
Influenza A(H1N1)pdm09^b^													
Overall	12	8–16		[11,27,31,32,36,38]	6	4–8	4	16	11–22	2	4	1–8	2
Outpatient	11	7–15		[11,31,36]	8	5–10	2	16	11–22	2	4	1–8	2
Inpatient	14	5–26		[27,32,38]	2	1–5	1	Na	Na	Na	Na	Na	Na
Influenza A(H3N2)													
Overall	11	8–14		[11,12,27,28,31-34,36,38,48]	7	4–9	11	8	4–12	3	10	2–22	4
Outpatient	13	9–17		[11,28,31,34,36,48]	10	7–13	10	8	4–12	3	10	2–22	4
Inpatient	9	5–14		[12,27,32,33,38]	4	1–8	1	Na	Na	Na	Na	Na	Na

ARI: acute respiratory infection; CI: confidence interval; ILI: influenza-like illness; Na: Not applicable; Ref: references; WHO: World Health Organization.

^a^ Pre-2009 pandemic seasons up to and including the 2008/09 season.

^b^ Post-pandemic seasons 2010/11 onwards.

For OPs, the following pooled estimates of proportion influenza positive patients were noted from studies which reported by age groups: 0–17 years, 26% (95% CI: 21–31, 18 studies, 32 datasets); 18–64 years, 41% (95% CI: 32–50, four studies, 14 datasets); and ≥65 years, 33% (95% CI: 27–40, five studies, 16 datasets) (Supplement S7). For IPs, data were only available for the age group 0–17 years, 9% (95% CI: 6–12, three studies, nine datasets) (Supplement S8).

The Table 1 shows the proportions of influenza A stratified into pre-pandemic A(H1N1), post-pandemic A(H1N1)pdm09, and A(H3N2) viruses, and influenza B in OPs and IPs across the different age groups. The highest proportion of influenza A infections was seen in the 18–64 age group with a pooled proportion estimate of 22% (95% CI: 16–29, four studies, 12 datasets), followed by the ≥65 years age group (18%; 95% CI: 14–31, four studies, 13 datasets). Similarly, the proportion infected with A(H1N1)pdm09 virus was highest in the age group 18–64 years at 16% (95% CI: 11–22, two studies, 10 datasets) in OPs. The highest proportion of influenza A(H3N2) virus was noted in the ≥ 65 years age group for 10% (95% CI: 2–22, four studies). For influenza B, the highest proportion of laboratory-confirmed patients were in the 18–64 year group; 15% (95% CI: 9–24, four studies), 12 datasets (Supplement S9 to S20).

Pooled estimates of the proportion of laboratory-confirmed influenza, stratified by influenza season, ranged from 19% in 2011/12 (95% CI: 9–31, two studies) and in 2013/14 (95% CI: 14–24, two studies, five datasets) to 48% (95% CI: 46–49, two studies) in 2002/03 (Supplement S21). When stratified by country, pooled estimates of laboratory-confirmed influenza ranged from 9% (two studies) to 65% (one study).

Stratification by the requirement for fever in the case definition of ARI/ILI in individual studies did not reveal a significant difference in the pooled proportion of influenza positivity between studies specifying the presence of fever and those in which it was not mandatory in 32% (95% CI: 25–39, eight studies) vs 31% (95% CI: 28–35, three studies respectively), p = 0.82). Heterogeneity was high (I^2^ > 90%) in all the meta-analyses.

### The European Surveillance System (TESSy) data

The ARI/ILI data were collected from 44 countries, while SARI data was collected from 15 countries (Supplement S2). Both datasets presented findings from persons of all ages that were not stratified by age group. The pooled estimate for all influenza in tested patients seeking medical attention from the ARI/ILI dataset was 33% (95% CI: 31–34, I^2^ = 99.4%), and for SARI the proportion was 24% (95% CI: 21–27, I^2^ = 98.8%). Table 2 shows the proportions of detections of influenza A, B, A(H1N1), A(H1N1)pdm09 and A(H3N2) viruses by case definition in TESSy.

**Table 2:** Pooled proportions of medically-attending patients with ARI/ILI/SARI testing positive for influenza from TESSy data by all influenza types and by influenza subtypes, WHO European Region, 2004 to 2017 (n = 670,550)

	Pooled proportion of influenza positive			
	ARI/ILI (OP: total tested 609,368)^a^		SARI (IP: total tested 61,182)^b^	
	%	95% Cl	%	95 Cl
All influenza	33	31–34	24	21–27
Influenza A	21	20–22	16	13–18
Influenza B	8	7–9	6	4–8
Influenza A(H1N1)				
Pre-pandemic	3	2–3	Na	Na
Post-pandemic	6	5–7	5	3–7
Influenza A(H3N2)	8	7–9	5	3–7

ARI: acute respiratory infection; CI: confidence interval; ILI: influenza-like illness; IP: inpatients; OP: outpatients; SARI: severe acute respiratory infection; WHO: World Health Organization. Denominator = number of specimens tested from patients with ARI/ILI/SARI.

^a ^44 countries, 558 country-years.

^b ^15 countries, 87 country-years.

**Figure 3:**
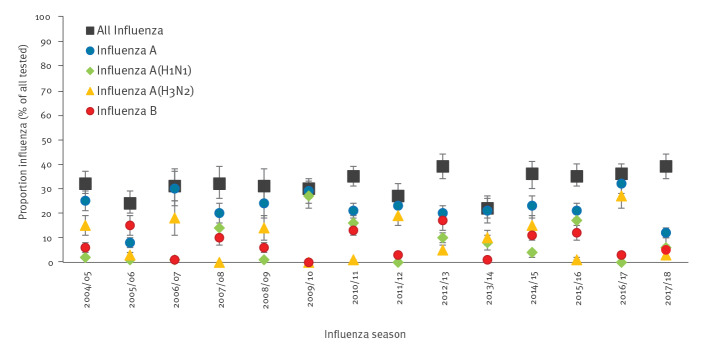
Influenza positivity as a proportion of all ARI/ILI tested by influenza season: all influenza and by type and subtype (TESSy data)

ARI: acute respiratory infection; ILI: influenza-like illness; TESSy: The European Surveillance System.

Pooled estimates of the proportion of laboratory-confirmed influenza in tested ARI/ILI patients for seasons between 2004/05 and between 2017/18, ranged from 22% (95% CI: 18–27) in 2013/14, to 39% (95% CI: 34–44) in 2012/13 and in 2017/18. The highest estimated proportions of laboratory-confirmed infections were noted in 2016/17 for influenza A with 32% (95% CI: 28–37); in 2015/16 for influenza A(H1N1)pdm09 with 17% (95% CI: 14–20) excluding the 2009/10 season; in 2016/17 for influenza A(H3N2) with 27% (95% CI: 22–32) and in 2012/13 for influenza B with 17% (95% CI: 13–21) (Figure 3 and Supplement S22).

The pooled estimated proportion of laboratory-confirmed influenza varied across countries, ranging from 6% (95% CI: 2–10, eight seasons) to 78% (one season) for ARI, and from 8% (one season) to 76% (one season) for SARI.

### Discussion

These are the most comprehensive data compiled to date on the proportion of laboratory-confirmed influenza across the WHO European Region in people presenting for medical care and with clinically-diagnosed ARI or ILI. We used two approaches: a literature review and a review of surveillance data, to estimate the prevalence of within-season influenza in the WHO European Region. Literature review data indicated that the proportion of seasonal influenza in OP was 36%, while the proportion in IPs was 24%. According to TESSy data, ARI/ILI proportion for all influenza types and subtypes was 33%, while the SARI proportion of all influenza was 24%. The 95% CI for the TESSy ARI/ILI and SARI data were narrower than those obtained from studies included in the literature review, reflecting the larger number of samples.

The lower proportion of laboratory-confirmed influenza in hospitalised patients compared with patients seeking outpatient care has also been found in other parts of the world [61-64]. Diagnostic tests for influenza perform best when specimens are collected as close to the onset of symptoms as possible, ideally within 72 h [65]. Hospitalised patients may experience symptoms for some time before admission and being swabbed which may decrease detection rate. The observation may also be a reflection of different characteristics of the OP and IP populations. In addition, neither the ARI/ILI nor the SARI case definition is specific to influenza.

Our pooled analyses estimates of the proportion of laboratory-confirmed influenza using both the literature review data and the TESSy data are somewhat higher than estimates in the US. A study pooling data from the North American literature estimated an influenza incidence proportion of 12% in children under 18 years (95% CI: 4.6–14.7) and 6.1% (95% CI: 4.3–7.9) in adults over several seasons when influenza severity was moderate [66]. However, there is some evidence that the situation in Europe may be different. In a meta-analysis examining the contribution of influenza to medically-attended ARI in children over several seasons in high-income countries, the proportion of influenza positive patients ranged from 18% in the US to 29% in Europe, which is closer to the results we obtained [15]. This may be partly explained by differences in influenza vaccine recommendations in Europe and the US. Although most countries in the European Union and European Economic Area have policies in place for seasonal influenza vaccination of people in high-risk groups, not all countries target children and vaccination coverage rates vary widely across the groups recommended for vaccination [67]. However, in the US seasonal influenza vaccination is recommended for everyone aged 6 months and older unless contraindicated [68]. Additionally, there may be differences between healthcare-seeking behaviour for ILI in different parts of the world, with some countries having a higher threshold than others [69].

The proportion of positive influenza tests varied from season to season in both the literature review results and in the analysis of the TESSy data. Over the totality of included seasons, influenza A accounted for a greater proportion of laboratory-confirmed influenza than influenza B. Since the 2009 pandemic, influenza A viruses have been dominant or co-dominant in seven of eight seasons across the WHO European Region. Influenza B-dominant seasons occur infrequently, most recently in 2017/18 [70]. Our literature review data of five seasons since the 2009 pandemic mirror these findings, with influenza A viruses accounting for the greatest proportion of laboratory-confirmed influenza in 2010/11, 2011/12, 2013/14 and 2014/15, and similar proportions of laboratory-confirmed influenza A and influenza B in 2012/13, a season when the influenza A and B viruses were co-dominant. Over the 10-year period between 1999 and the 2009 pandemic, seven influenza seasons in Europe were dominated by influenza A(H3N2) viruses, with or without co-circulation of influenza B viruses. Notable circulation of pre-pandemic A(H1N1) viruses occurred in only two of these seasons, in 2000/01 and 2007/08 [71-74], which is in accordance with the data from both our literature review and the TESSY analysis.

We acknowledge that our study has a number of limitations. Observed differences in the estimated proportions of influenza-positive patients between age groups should be interpreted with caution, since these may arise through variation in healthcare seeking behaviour according to age. There is evidence that younger working age adults are less likely to seek healthcare than children and even then, only when they are very unwell, so the denominator may be smaller in this age group which could lead to increased overall positivity in accordance with our findings [75]. We were only able to collect age group data from the literature review, and the papers identified varied in how age was categorised. There were few studies that reported specifically on children who were under 3 years old. Most included studies reported on the age group ranging from 0 to 17 years, therefore it is possible that older children have been over-represented in our meta-analysis. Furthermore, although we did not include data for the 2009 pandemic, it is possible that health-seeking behaviour may have increased in some age groups in the seasons immediately following the pandemic and this may have affected our findings.

We defined influenza burden as the percentage of patients with an ARI or ILI seeking medical care and tested for respiratory viruses, who have laboratory-confirmed influenza. We are unable to comment on the burden of laboratory-confirmed influenza in people with ARI or ILI who seek care but are not tested, or in those who develop symptoms but do not seek medical care. The proportion of true influenza in each of these populations may well be lower than our estimates. A study from the US estimated that 38% of people with influenza present for medical care. This proportion is lower than the proportion of people with respiratory syncytial virus or adenovirus infections who present for medical care but greater than in those with rhinovirus, coronavirus, parainfluenza virus and other respiratory viral infections [76]. A FluWatch cohort study over five seasons from the UK found that only 17% of those with PCR-confirmed influenza had medically-attended illness [1]. Other studies from Europe have shown that the majority of people with ARI or ILI do not seek medical care, but with much variation between countries and between northern and southern Europe, suggesting regional cultural differences [69]. Healthcare seeking behaviour and clinician behaviour are complex issues and decisions made at different points of the clinical interaction may affect the overall composition of the sampled population in terms of severity of illness. Even if influenza attack-rates and healthcare seeking behaviour are similar between countries, if the propensity of clinicians to test patients, refer them to hospital and to admit them differs across countries, the resulting hospitalised populations will vary in the severity of their illness. Since influenza shows clear seasonality in temperate regions, our estimates from the literature review and from TESSy were within-season estimates, so the positivity estimates do not apply to respiratory illnesses occurring outside the influenza season. This also limits direct comparability to existing influenza burden estimates, including multiplier-based burden analyses and global burden estimates, which use annualised estimates and may thus have lower influenza positivity [19,77,78]. However, within-season estimates have more relevance for public health planning in countries with defined influenza seasons.

In the literature review, one expert reviewed the references and extracted the data, so it is possible that some studies were missed. Additionally, there was greater representation of countries in the western part of Europe in the published literature. Although we did not exclude non-English language studies, it is possible that the database searched was less likely to include studies from countries in eastern Europe. We did not search Russian language databases in our literature review which could have added more data from eastern European countries.

Heterogeneity was high in the meta-analysis, but as our outcomes were absolute measures rather than ratio measures which tend to be more stable across studies, this was not unexpected [79]. Multiple factors are also likely to cause such heterogeneity, including differences in healthcare systems, case definitions, age groups, climate, vaccination coverage and general health, which makes comparisons challenging. It is also likely that there are cultural differences between countries in terms of the healthcare-seeking behaviour. We included papers reporting ARI or ILI as defined by the individual studies rather than standardised definitions, so this is an additional potential source of heterogeneity. In 2011, the WHO revised the clinical case definition of ILI to enhance its specificity without greatly compromising its sensitivity, such that the requirement for ‘sore throat’ and ‘absence of another diagnosis’ were omitted, and ‘sudden onset of fever’ was replaced by ‘acute respiratory illness’. The case definition of an ARI does not require fever to be present, so it is less specific for detecting influenza than the revised ILI definition [80]. We explored potential sources of heterogeneity through stratification and sensitivity analyses, yet considerable heterogeneity remained, and the results should be interpreted taking this unexplained heterogeneity into consideration.

Notwithstanding, we believe that this study adds to the knowledge base on the contribution of seasonal influenza virus infections to respiratory illness across the WHO European Region. Estimates of influenza positivity can help with appropriate allocation of limited health resources among competing disease priorities, establish epidemic thresholds for comparison of disease severity between seasons and localities, and provide a platform for the evaluation of the effectiveness of vaccines and other interventions [81]. Particular strengths of this study include the use of viral culture or RT-PCR, which is the gold standard for influenza diagnosis because of its superior analytic and clinical sensitivity [82]. In the literature review there was careful consideration of the inclusion criteria and a risk of bias assessment was undertaken for each eligible study. Additionally, we relied on data collected individually within a full season which strengthens the validity of our results.

## Conclusion

This analysis estimated the proportion of laboratory-confirmed seasonal influenza in symptomatic people who presented for healthcare with ARI/ILI and were subsequently tested for influenza viruses in the WHO European Region across the influenza seasons between 1996 and 2017. The estimated proportion of positive tests was shown to be greater in OP than in hospitalised patients by both methods, with differences according to influenza subtype and across different age groups. Overall, in Europe, laboratory-confirmed influenza accounts for around approximately one third of all acute respiratory infections for which medical care is sought during the influenza season and where laboratory testing for influenza is undertaken. The effect of the ongoing COVID-19 pandemic on healthcare-seeking behaviour for ILI, and changes in countries’ testing priorities and capacities, may potentially affect estimates of influenza positivity in future seasons. This should be taken into account when comparing our results to those of future studies and will require further investigation in forthcoming influenza seasons.

## Supplementary Data

629000 Supplementary Material
