# Supplementary material for: Spotlight influenza: Laboratory-confirmed seasonal influenza in people with acute respiratory illness: a literature review and meta-analysis, WHO European Region, 2004 to 2017
Source: Euro Surveill. 2021 Sep 30;26(39):2000343. doi: 10.2807/1560-7917.ES.2021.26.39.2000343 (PMC8485580; doi:10.2807/1560-7917.ES.2021.26.39.2000343)
Supplement: Supplementary Material [file Supplement_20_00343_LANSBURY.pdf]

This supplementary material is hosted by *Eurosurveillance* as supporting information alongside the article “Laboratory-confirmed seasonal influenza in people with acute respiratory illness: a literature review and meta-analysis, WHO European Region, 2004 to 2017” on behalf of the authors who remain responsible for the accuracy and appropriateness of the content. The same standards for ethics, copyright, attributions and permissions as for the article apply. Supplements are not edited by Eurosurveillance and the journal is not responsible for the maintenance of any links or email addresses provided therein.

S1: Medline Search Strategy

1. Influenza.mp. or exp Influenza, Human/
2. exp Pneumonia/ or pneumonia.mp. or exp Pneumonia, Viral/
3. community acquired pneumonia.mp.
4. exp Respiratory Tract Infections/ or acute respiratory infection.mp.
5. respiratory tract illness.mp.
6. (influenza-like illness or influenza like illness or ARI or SARI or ILI).mp. [mp=title, abstract, original title, name of substance word, subject heading word, keyword heading word, protocol supplementary concept word, rare disease supplementary concept word, unique identifier, synonyms]
7. incidence.mp. or exp Incidence/
8. exp Prevalence/ or prevalence.mp.
9. exp Hospital Mortality/ or exp Mortality/ or exp Child Mortality/ or exp Maternal Mortality/ or Mortality.mp. or exp Infant Mortality/
10. death.mp. or exp "Cause of Death"/ or exp Death/
11. morbidity.mp. or exp Morbidity/
12. (burden or impact).mp.
13. epidemiology.mp. or exp Epidemiology/
14. exp Hospitalization/ or hospitalisation.mp. or exp "Length of Stay"/
15. 1 or 2 or 3 or 4 or 5 or 6
16. 7 or 8 or 9 or 10 or 11 or 12 or 13 or 14
17. 15 and 16
18. limit 17 to (humans and yr="2015 -Current")

S2: TESSy participating countries and duration of participation

Country	First season of data contributing to review	
	ARI/ILI	SARI
Albania	2007-2008	2011-2012
Armenia	2010-2011	2009-2010
Austria	2004-2005	-
Azerbaijan	2008-2009	2015-2016
Belarus	2006-2007	2015-2016
Belgium	2004-2005	-
Bulgaria	2007-2008	-
Croatia	2013-2014	-
Czech Republic	2004-2005	-
Denmark	2004-2005	-
Estonia	2005-2006	-
Finland	2004-2005	-
France	2004-2005	-
Georgia	2008-2009	2010-2011
Germany	2004-2005	-
Greece	2004-2005	-
Hungary	2004-2005	-
Ireland	2004-2005	-
Israel	2004-2005	-
Italy	2004-2005	-
Kazakhstan	2008-2009	2009-2010
Kyrgyzstan	2008-2009	2009-2010
Latvia	2004-2005	-
Lithuania	2004-2005	-
Luxembourg	2004-2005	-
North Macedonia	2016-2017	-
Malta	2010-2011	-
Netherlands	2004-2005	-
Norway	2004-2005	-
Poland	2004-2005	-
Portugal	2004-2005	-
Republic of Moldova	2009-2010	2010-2011
Romania	2004-2005	-
Russian Federation	2009-2010	2010-2011
Serbia	2006-2007	2012-2013
Slovakia	2004-2005	-
Slovenia	2004-2005	-
Spain	2004-2005	-
Sweden	2006-2007	-
Switzerland	2004-2005	-
Tajikistan	2009-2010	2017-2018
Turkey	2008-2009	-
United Kingdom	2004-2005	-
Ukraine	2006-2007	2009-2010
Uzbekistan	2012-2013	2017-2018

S3: Studies included in meta-analysis

(1) Fajfr M, Stěpánová V, Plíšková L. Influenza in seasons 2009-2013 in the faculty Hospital Hradec kralove, East Bohemia. Epidemiol Mikrobiol Imunol 2014;63(1):6-10.

(2) Pierangeli A, Gentile M, Di Marco P, Pagnotti P, Scagnolari C, Trombetti S et al. Detection and typing by molecular techniques of respiratory viruses in children hospitalized for acute respiratory infection in Rome, Italy. J Med Virol 2007;79(4):463-468.

(3) Puig-Barberà J, Tormos A, Sominina A, Burtseva E, Launay O, Ciblak M et al. First-year results of the Global Influenza Hospital Surveillance Network: 2012–2013 Northern hemisphere influenza season. BMC Public Health 2014;14(1).

(4) Redlberger-Fritz M, Aberle J, Popow-Kraupp T, Kundi M. Attributable deaths due to influenza: a comparative study of seasonal and pandemic influenza. Eur J Epidemiol 2012;27(7):567-575.

(5) Rezza G, Valdarchi C, Puzelli S, Ciotti M, Farchi F, Fabiani C et al. Respiratory viruses and influenza-like illness: a survey in the area of Rome, winter 2004-2005. Euro Surveill 2006;11(10):9-10.

(6) Meerhoff T, Simaku A, Ulqinaku D, Torosyan L, Gribkova N, Shimanovich V et al. Surveillance for severe acute respiratory infections (SARI) in hospitals in the WHO European region - an exploratory analysis of risk factors for a severe outcome in influenza-positive SARI cases. BMC Infect Dis 2015;15(1).

(7) Harvala H, Smith D, Salvatierra K, Gunson R, von Wissmann B, Reynolds A et al. Burden of influenza B virus infections in Scotland in 2012/13 and epidemiological investigations between 2000 and 2012. Euro Surveill 2014;19(37).

(8) Pebody R, Warburton F, Ellis J, Andrews N, Thompson C, von Wissmann B et al. Low effectiveness of seasonal influenza vaccine in preventing laboratory-confirmed influenza in primary care in the United Kingdom: 2014/15 mid–season results. Euro Surveill 2015;20(5).

(9) Puig-Barberà J, Natividad-Sancho A, Trushakova S, Sominina A, Pisareva M, Ciblak M et al. Epidemiology of Hospital Admissions with Influenza during the 2013/2014 Northern Hemisphere Influenza Season: Results from the Global Influenza Hospital Surveillance Network. PLoS One 2016;11(5):e0154970.

(10) Puig-Barberà J, Burtseva E, Yu H, Cowling B, Badur S, Kyncl J et al. Influenza epidemiology and influenza vaccine effectiveness during the 2014–2015 season: annual report from the Global Influenza Hospital Surveillance Network. BMC Public Health 2016;16(S1).

(11) Redlberger-Fritz M, Kundi M, Popow-Kraupp T. Detailed Report on 2014/15 Influenza Virus Characteristics, and Estimates on Influenza Virus Vaccine Effectiveness from Austria's Sentinel Physician Surveillance Network. PLoS One 2016;11(3):e0149916.

(12) Plymoth A, Rotzen-Ostlund M, Zweygberg-Wirgart B, Sundin CG, Ploner A, Nyren O, et al. Self-sampling for analysis of respiratory viruses in a large-scale epidemiological study in Sweden. Euro Surveill 2015;20(11):19.

(13) an der Heiden M, Buchholz U. Estimation of influenza-attributable medically attended acute respiratory illness by influenza type/subtype and age, Germany, 2001/02-2014/15. Influenza Other Respir Viruses 2016;11(2):110-121.

(14) Mosnier A, Caini S, Daviaud I, Bensoussan J, Stoll-Keller F, Bui T et al. Ten influenza seasons in France: distribution and timing of influenza A and B circulation, 2003–2013. BMC Infect Dis 2015;15(1).

(15) Boddington N, Verlander N, Pebody R. Developing a system to estimate the severity of influenza infection in England: findings from a hospital-based surveillance system between 2010/2011 and 2014/2015. Epidemiol Infect 2017;145(07):1461-1470.

(16) Beauté J, Zucs P, Korsun N, Bragstad K, Enouf V, Kossyvakis A et al. Age-specific differences in influenza virus type and subtype distribution in the 2012/2013 season in 12 European countries. Epidemiol Infect 2015;143(14):2950-2958.

(17) Gasparini R, Durando P, Ansaldi F, Sticchi L, Banfi F, Amicizia D et al. Influenza and respiratory syncytial virus in infants and children: relationship with attendance at a paediatric emergency unit and characteristics of the circulating strains. Eur J Clin Microbiol Infect Dis 2007;26(9):619-628.

(18) Gooskens J, van der Ploeg V, Sukhai R, Vossen A, Claas E, Kroes A. Clinical evaluation of viral acute respiratory tract infections in children presenting to the emergency department of a tertiary referral hospital in the Netherlands. BMC Pediatr 2014;14(1).

(19) Esposito S, Cantarutti L, Molteni C, Daleno C, Scala A, Tagliabue C et al. Clinical manifestations and socio-economic impact of influenza among healthy children in the community. J Infect 2011;62(5):379-387.

(20) Esposito S, Molteni C, Daleno C, Valzano A, Fossali E, Da Dalt L et al. Clinical and socioeconomic impact of different types and subtypes of seasonal influenza viruses in children during influenza seasons 2007/2008 and 2008/2009. BMC Infect Dis 2011;11(1).
(21) Esposito S, Gasparini R, Bosis S, Marchisio P, Tagliabue C, Tosi S et al. Clinical and socio–economic impact of influenza and respiratory syncytial virus infection on healthy children and their households. Clin Microbiol Infect 2005;11(11):933-936.
(22) Ajayi-Obe E, Coen P, Handa R, Hawrami K, Aitken C, McIntosh E et al. Influenza A and respiratory syncytial virus hospital burden in young children in East London. Epidemiol Infect 2007;136(08).
(23) Paixão P, Piedade C, Papoila A, Caires I, Pedro C, Santos M et al. Improving influenza surveillance in Portuguese preschool children by parents' report. Eur J Pediatr 2014;173(8):1059-1065.
(24) Tsolia M, Logotheti I, Papadopoulos N, Mavrikou M, Spyridis N, Drossatou P et al. Impact of influenza infection in healthy children examined as outpatients and their families. Vaccine. 2006;24(33-34):5970-5976.
(25) Kouni S, Karakitsos P, Chranioti A, Theodoridou M, Chrousos G, Michos A. Evaluation of viral co-infections in hospitalized and non-hospitalized children with respiratory infections using microarrays. Clin Microbiol Infect 2013;19(8):772-777.
(26) Pebody R, Andrews N, McMenamin J, Durnall H, Ellis J, Thompson C et al. Vaccine effectiveness of 2011/12 trivalent seasonal influenza vaccine in preventing laboratory-confirmed influenza in primary care in the United Kingdom: evidence of waning intra-seasonal protection. Euro Surveill 2013;18(5).
(27) Karadag-Oncel E, Ciblak M, Ozsurekci Y, Badur S, Ceyhan M. Viral etiology of influenza-like illnesses during the influenza season between December 2011 and April 2012. J Med Virol 2013;86(5):865-871.
(28) Castilla J, Guevara M, Martínez-Baz I, Ezpeleta C, Delfrade J, Irisarri F et al. Enhanced Estimates of the Influenza Vaccination Effect in Preventing Mortality. Medicine (Baltimore) 2015;94(30):e1240.
(29) Chatzopoulou E, Melidou A, Gioula G, Exindari M, Chatzidimitriou D, Chatzopoulou F et al. Contribution of influenza viruses, human metapneumovirus and respiratory syncytial virus to acute respiratory infections in children in northern Greece, 2008 – 2010. Eastern J Med. 2012; 17(1): 24-29
(30) Principi N, Esposito S, Marchisio P, Gasparini R, Crovari P. Socioeconomic impact of influenza on healthy children and their families. Pediatr Infect Dis J 2003;22(10 Suppl):S207-10.
(31) Meury S, Zeller S, Heininger U. Comparison of clinical characteristics of influenza and respiratory syncytial virus infection in hospitalised children and adolescents. Eur J Pediatr 2004;163(7).
(32) Heikkinen T, Silvennoinen H, Peltola V, Ziegler T, Vainionpaa R, Vuorinen T, et al. Burden of influenza in children in the community. J Infect Dis 2004;190(8):1369-73.
(33) Bennet R, Hamrin J, Wirgart B, Östlund M, Örtqvist Å, Eriksson M. Influenza epidemiology among hospitalized children in Stockholm, Sweden 1998–2014. Vaccine 2016;34(28):3298-3302.
(34) Tsolia M, Psarras S, Bossios A, Audi H, Paldanius M, Gourgiotis D et al. Etiology of Community-Acquired Pneumonia in Hospitalized School-Age Children: Evidence for High Prevalence of Viral Infections. Clin Infect Dis 2004;39(5):681-686.
(35) Heikkinen T, Ziegler T, Peltola V, Lehtinen P, Toikka P, Lintu M, et al. Incidence of influenza in Finnish children. Pediatr Infect Dis J 2003;22(10 Suppl):S204-6.
(36) Silvennoinen H, Peltola V, Lehtinen P, Vainionpää R, Heikkinen T. Clinical Presentation of Influenza in Unselected Children Treated as Outpatients. Pediatr Infect Dis J 2009;28(5):372-375.
(37) Zambon MC, Stockton JD, Clewley JP, Fleming DM. Contribution of influenza and respiratory syncytial virus to community cases of influenza-like illness: an observational study. Lancet 2001;358(9291):1410-6
(38) Zieliński A, Czarkowski M, Sadkowska-Todys M. Infectious Diseases in Poland in 2011. Przegl Epidemiol 2013;67:171 - 179.

S4: Sensitivity Analysis: RT-PCR confirmed influenza only (omitting studies in which influenza only confirmed by viral culture/IF)

Influenza virus	Pooled estimate of proportion positive %	% change from original estimate	Pooled estimate of proportion positive by age group (%) (95% CI, number of studies)					
			0-17yrs	% change	18-64yrs	% change	>65 yrs	% change
**Any influenza virus**								
Outpatient	36	n/a	26	0	41	n/a	33	n/a
Inpatient	24	n/a	9	n/a	-	-	-	-
**Influenza A**								
Outpatient	25	+1	14	n/a	22	n/a	18	n/a
Inpatient	20	n/a	6	n/a	-	-	-	-
**Influenza B**								
Outpatient	7	-2	9	+1	15	n/a	10	n/a
Inpatient	5	+1	3	n/a	-	-	-	-
**Influenza A(H1N1): pre 2009 pandemic**								
Outpatient	3	n/a	3	n/a	-	-	-	-
Inpatient	-	-	-	-	-	-	-	-
**Influenza A(H1N1): post 2009 pandemic**								
Outpatient	11	n/a	8	n/a	16	n/a	4	n/a
Inpatient	14	n/a	2	n/a	-	n/a	-	-
**Influenza A(H3N2)**								
Outpatient	13	n/a	9	-1	8	n/a	10	n/a
Inpatient	9	n/a	3	-1	-	-	-	n/a

n/a not applicable, no studies excluded in sensitivity analysis; - No data

Studies excluded in sensitivity analysis**:** Tsolia 2006, Bennet 2016, Silvennoinen 2009, Heikkinen 2003, Heikkinen 2004, Principi 2004

S5: Definitions of quality levels and numbers of studies included in meta-analyses

Quality indicator	Study Quality Level		
	**High**	**Intermediate**	**Low**
Geographic representativeness	Multi-city study<br>(N=18)	Multiple centres in one city<br>(N= 6)	One centre in a single city<br>(N=16)
Age representativeness	Includes well defined and specified age groups from an entire child (0-17 years, 18-year span) to an adult (18-64 years, 47-year span) age span<br>(N= 7)	One or two well defined age groups<br>(N=23)	Age span not specified or includes <9 years for children or <23.5 years for adults<br>(N= 10)
General representativeness	Randomly or systematically chosen subjects<br>(N=2)	General population volunteers may exclude those with high risk of influenza complications<br>(N=38)	Includes only members of a specific group (e.g., college students)<br>(N= 0)
Sensitivity of symptoms prompting laboratory testing	Does not require any one specific symptom<br>(N=24)	Requires one specific symptom; or includes URI without listing specific symptoms<br>(N= 8)	Requires ≥2 specific symptoms<br><br>(N= 8)
Laboratory method	RT-PCR with or without culture<br>(N=35)	Viral culture without RT-PCR<br>(N=5)	Methods other than RT-PCR or culture<br>(N=0)

S6: Quality levels for studies included in meta-analyses

First author, year published	Age-groups(years)	Number seasons	Geographic representativeness	Age representativeness	General representativeness	Sensitivity of symptoms	Laboratory method
Gasparini 2007	0-19, 0-3, 3-19	2	Low	Intermediate	Intermediate	Intermediate	High
Gooskens 2014	0-3, 3-19,0-19	1	Low	Intermediate	Intermediate	High	High
Esposito 2011 c	0-19	1	High	Intermediate	Intermediate	High	High
Esposito 2011b	0-19	2	High	Intermediate	Intermediate	High	High
Esposito 2005	0-19	1	Low	Intermediate	Intermediate	High	High
Silvennoinen 2009	0-19	2	Low	Intermediate	Intermediate	High	Intermediate
Ajayi-Obe 2008	0-7	2	Low	Intermediate	Intermediate	High	High
Paixao 2014	0-7	1	Intermediate	Intermediate	Intermediate	Low	High
Plymoth 2015	15-44, 45-65	2	Intermediate	High	High	Intermediate	High
Kouni 2013	0-19	1	Low	Intermediate	Intermediate	Intermediate	High
Pebody 2013	0-7,3-19, 15-44 ,45-64, ≥65, all	1	High	High	Intermediate	High	High
Meerhoff 2015	0-19, all	3	High	Intermediate	Intermediate	High	High
Tsolia 2006	3-19	2	High	Intermediate	Intermediate	Low	Intermediate
Harvala 2014	0-7, 3-19, 15-44, 37-65, ≥65, all	1	High	High	Intermediate	High	High
Heikkinen 2004	0-3, 0-7, 3-19, 0-19	2	Intermediate	Intermediate	Intermediate	High	Intermediate
Karadag-oncel 2014	0-19	1	Low	Intermediate	Intermediate	Low	High
Pebody 2015	0-19, 15-44, 45-64, ≥65, all	1	High	High	Intermediate	High	High
Castilla 2015	≥65	2	Low	Intermediate	Intermediate	Intermediate	High
Chatzopoulou 2012	0-7	1	Intermediate	Intermediate	Intermediate	Intermediate	High
Principi 2004	0-19	1	High	Intermediate	Intermediate	Low	Intermediate
Meury 2004	0-19y	2	Low	Intermediate	Intermediate	High	High
Beaute 2015	0-7, 3-19, 45-65, ≥65,all	1	High	High	Intermediate	High	High
Zambon 2001	0-7,3-19,15-44, 45-65, 65,all	3	High	High	Intermediate	High	High
Heikkinen 2003	0-19	1	Low	Intermediate	Intermediate	High	Intermediate
Bennet 2016	0-19	6	Low	Intermediate	Intermediate	Intermediate	Intermediate
Tsolia 2004	3-19	1	Low	Intermediate	Intermediate	Low	High
Pierangeli 2007	All	1	Low	Intermediate	Intermediate	High	High
Redlberger-Fritz 2012	All	10	Intermediate	High	Intermediate	Low	High
Zielinski 2013	All	1	High	Low	Intermediate	High	High
Rezza 2006	All	1	Intermediate	Low	Intermediate	Low	High
Mosnier 2015	All	9	High	Low	Intermediate	Intermediate	High
Puig- Barbera 2014	All	1	High	Low	Intermediate	High	High
Puig-Barbara 2015	All	1	High	Low	Intermediate	High	High
Puig-Barbara 2016	All	1	High	Low	Intermediate	High	High
Redlberger-Fritz 2016	All	1	High	Low	Intermediate	High	High

Boddington 2017	All	4	High	Low	High	Low	High
Der Heidan 2016	All	13	High	Low	Intermediate	Intermediate	High
Fajfr 2014	All	3	Low	Low	Intermediate	High	High

S7: Forest plot (studies from literature review): proportion of all influenza viruses by age groups in outpatients seeking care in Europe

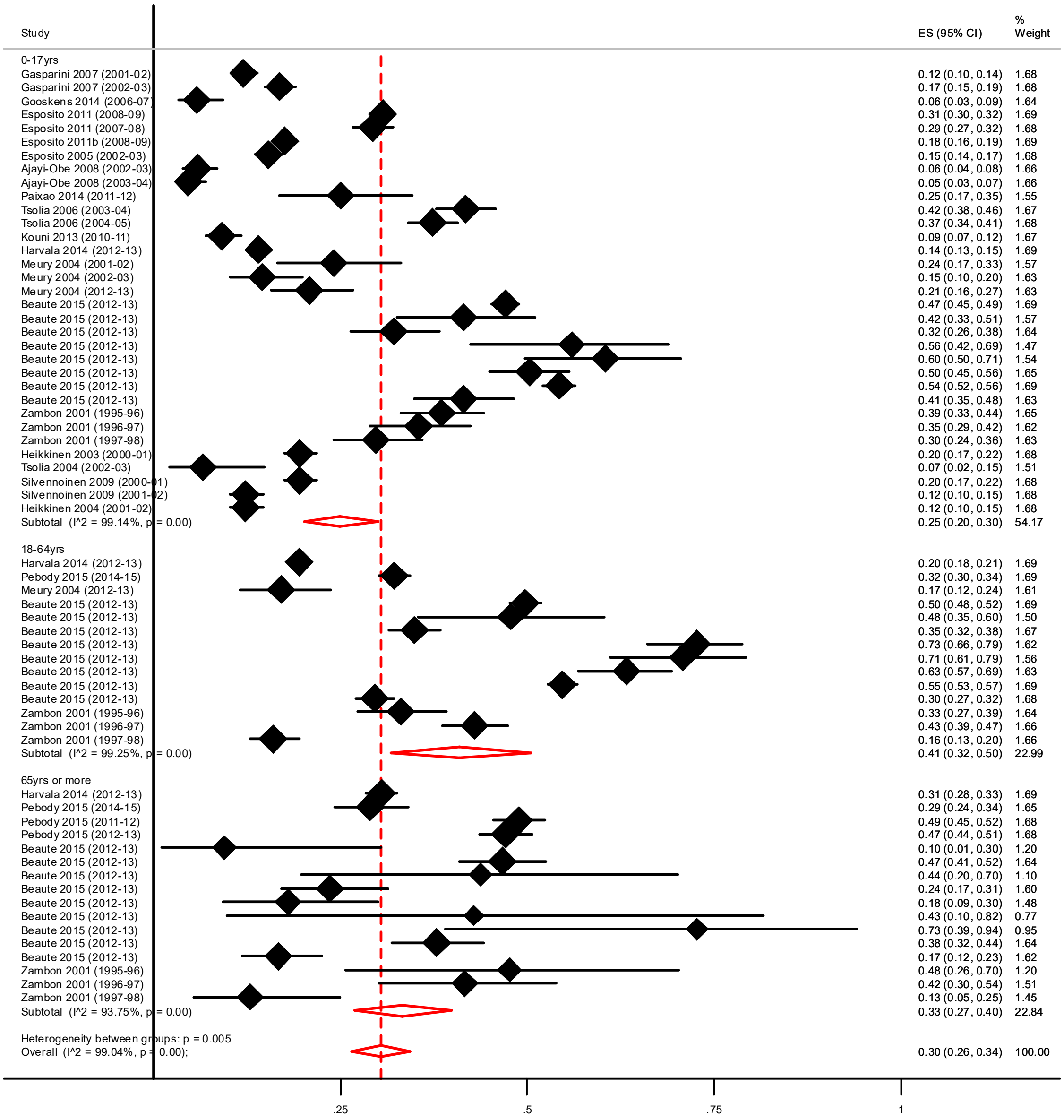

S8: Forest plot (studies from literature review): proportion of all influenza viruses by age groups in inpatients seeking care in Europe

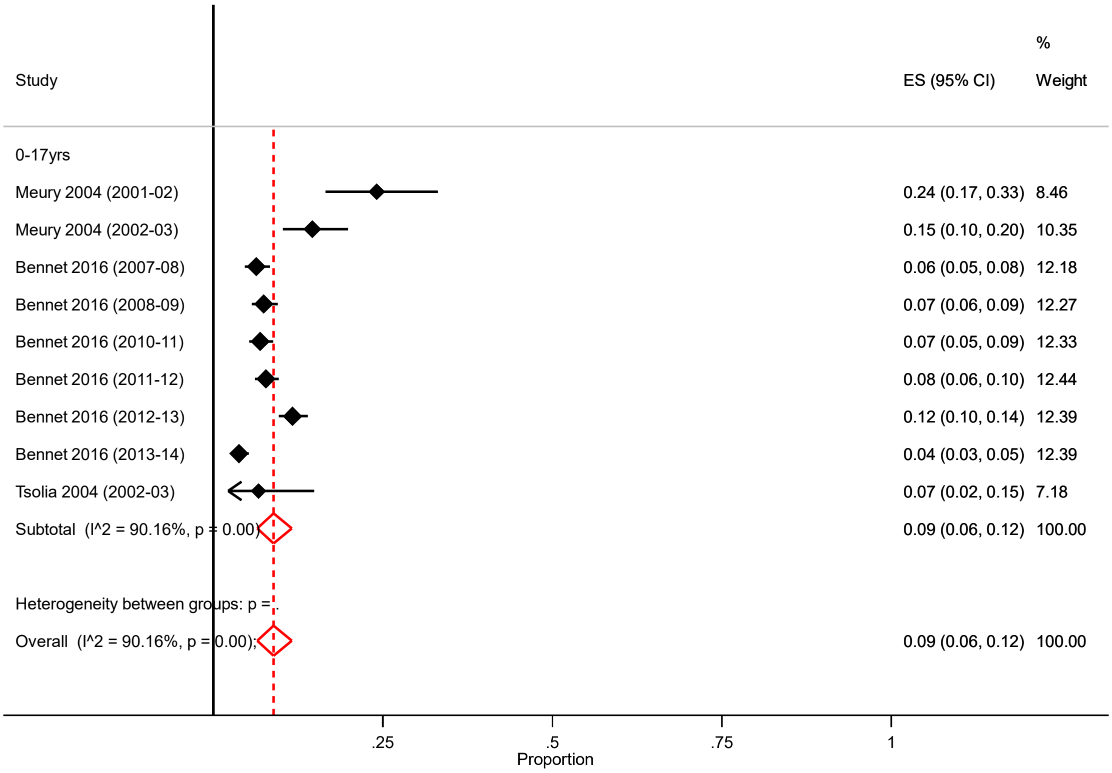

S9: Forest plot (studies from literature review): proportion of influenza A virus subgroup analysis by healthcare setting

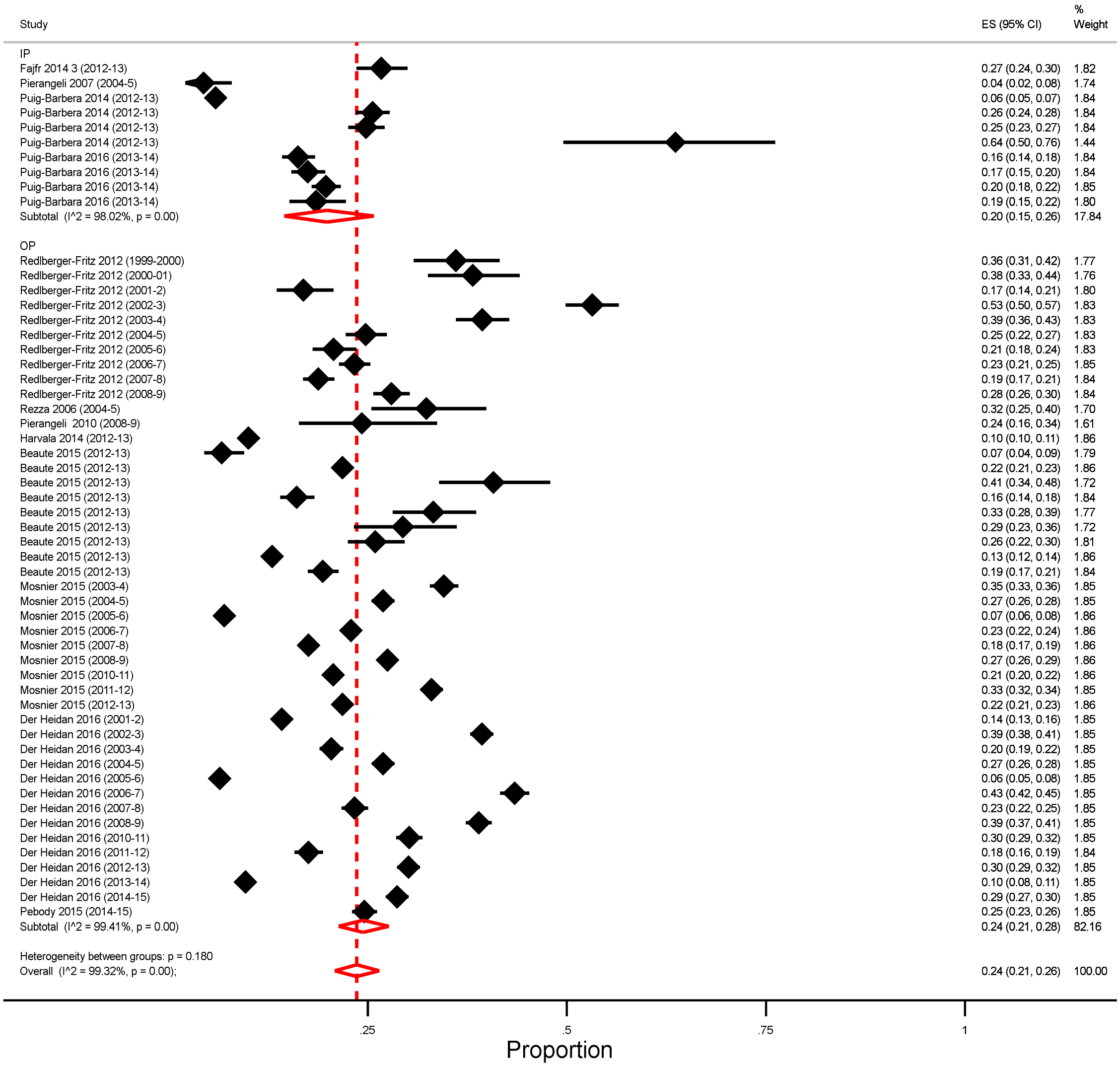

S10: Forest plot (studies from literature review): proportion of influenza A viruses by age groups in outpatients seeking care in Europe

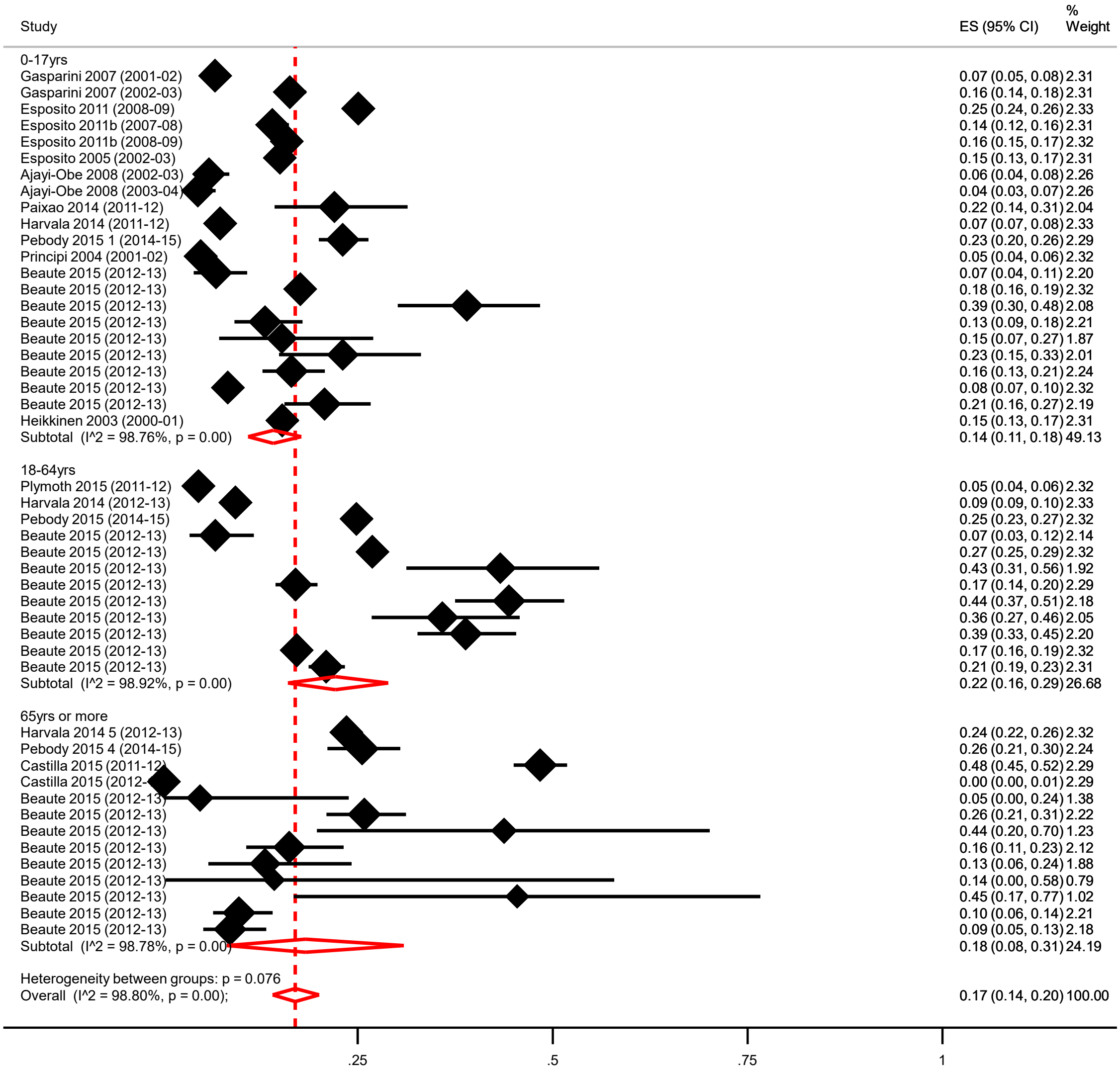

S11: Forest plot (studies from literature review): proportion of influenza A viruses by age groups in Inpatients seeking care in Europe

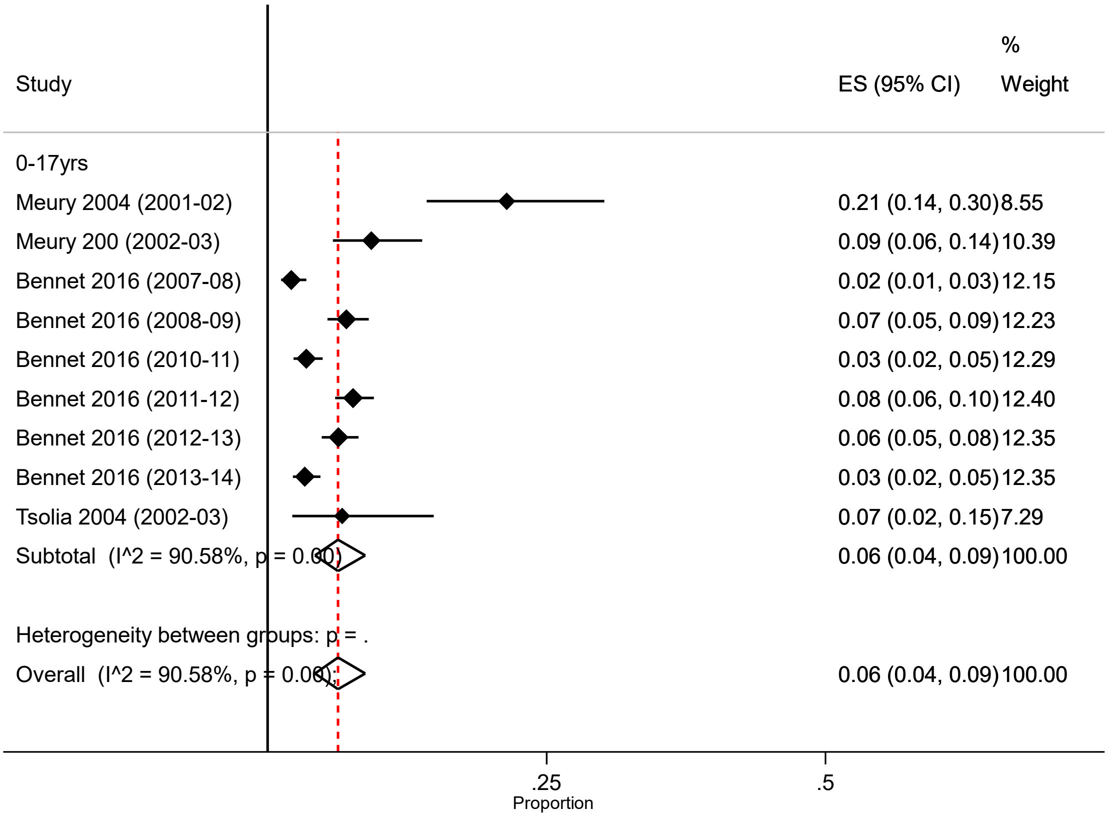

S12: Forest plot (studies from literature review): proportion of influenza B viruses subgroup analysis by healthcare setting in patients seeking care in Europe

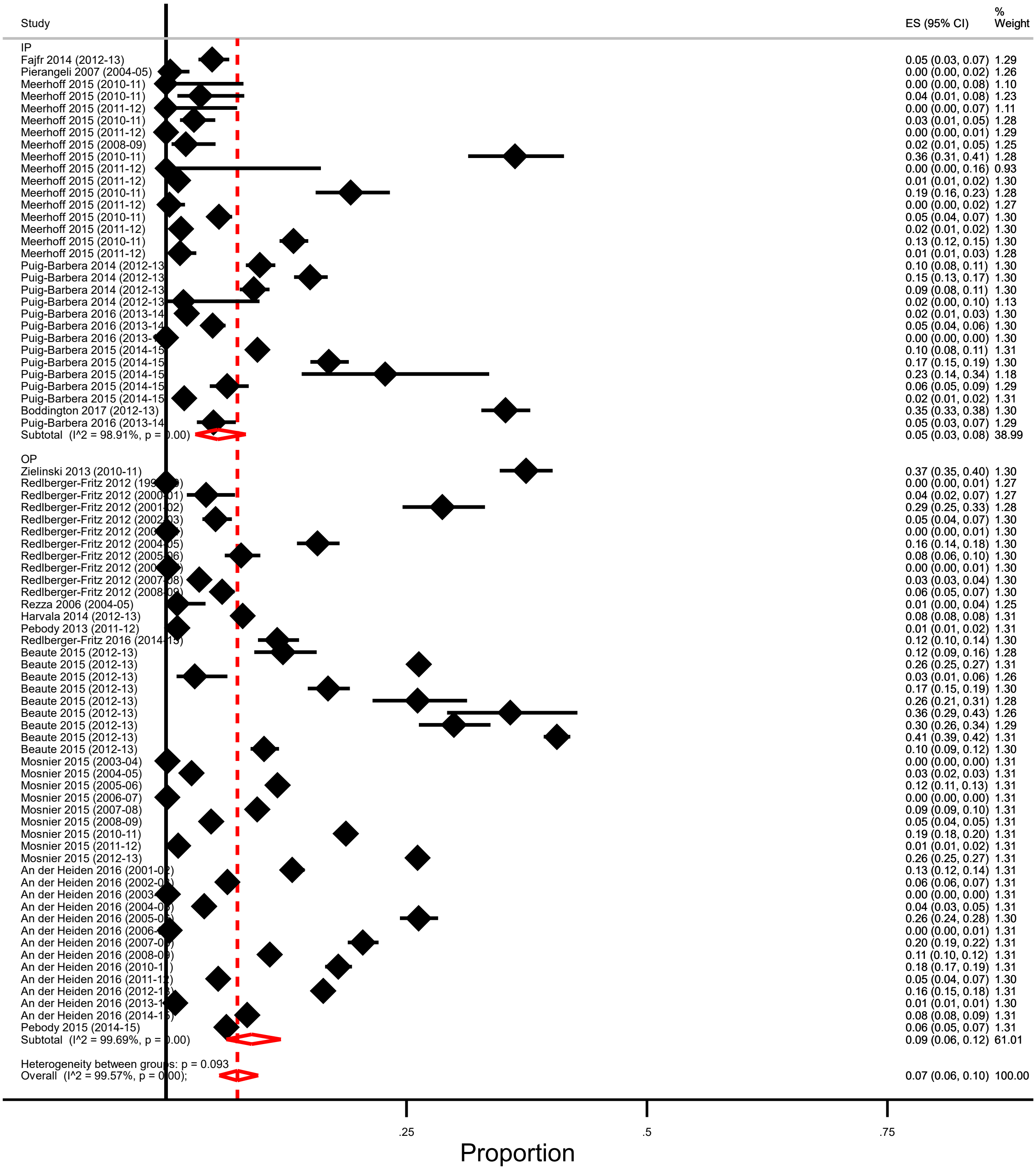

S13: Forest plot (studies from literature review): proportion of influenza B viruses by age groups in outpatients seeking care in Europe

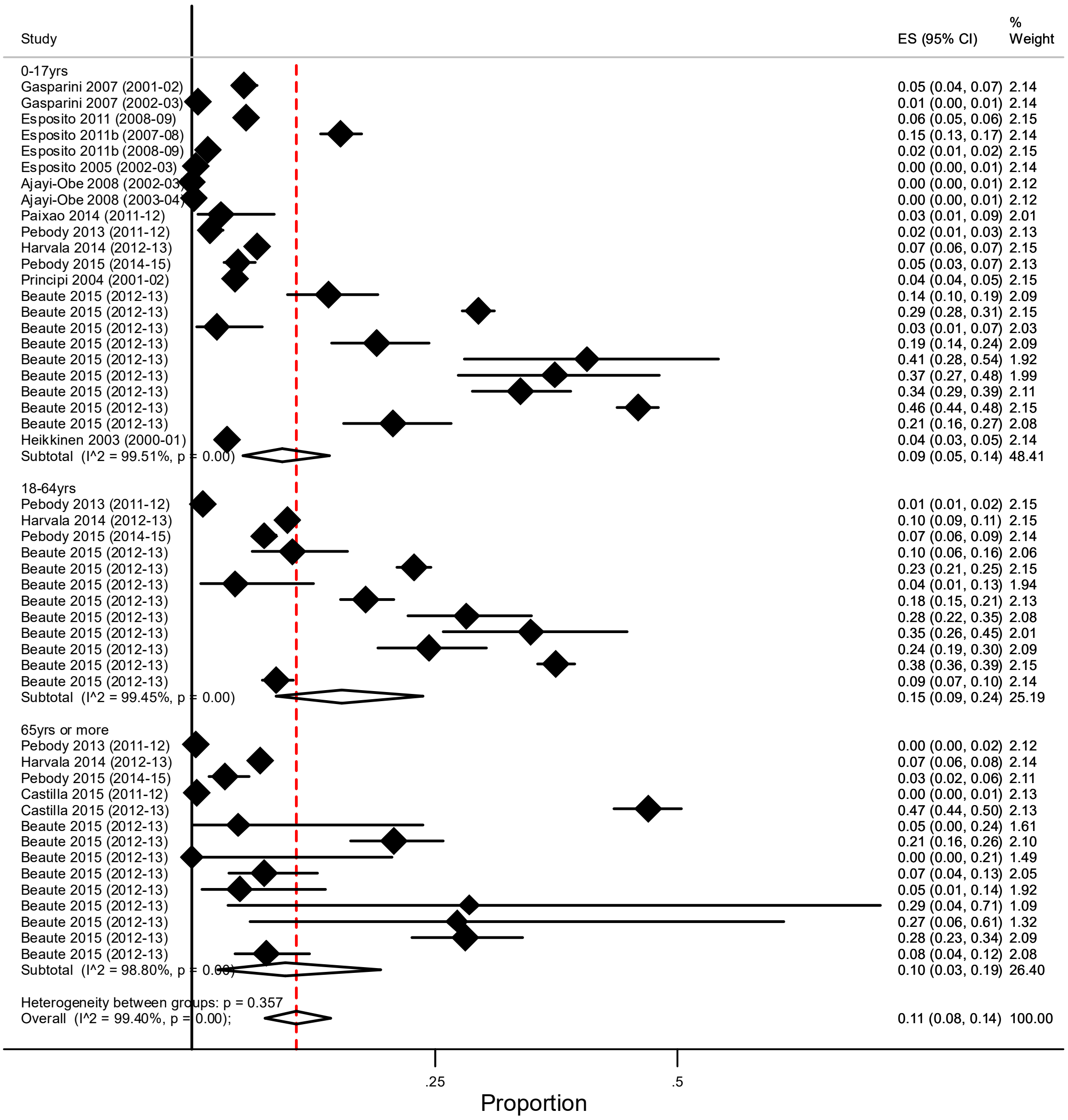

S14: Forest plot (studies from literature review): proportion of influenza B viruses by age groups in Inpatients seeking care in Europe

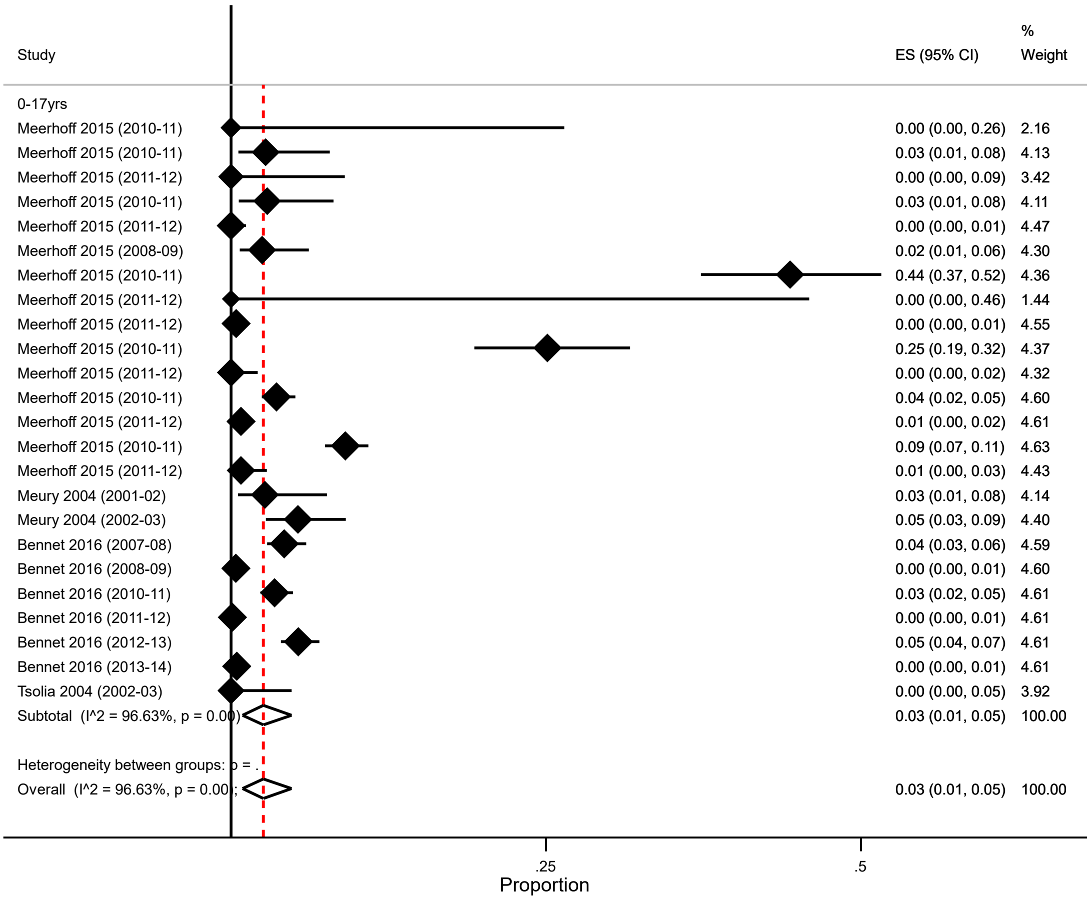

S15a: Forest plot (studies from literature review): proportion of pre-pandemic influenza A(H1N1) viruses subgroup analysis by healthcare setting in patients seeking care in Europe

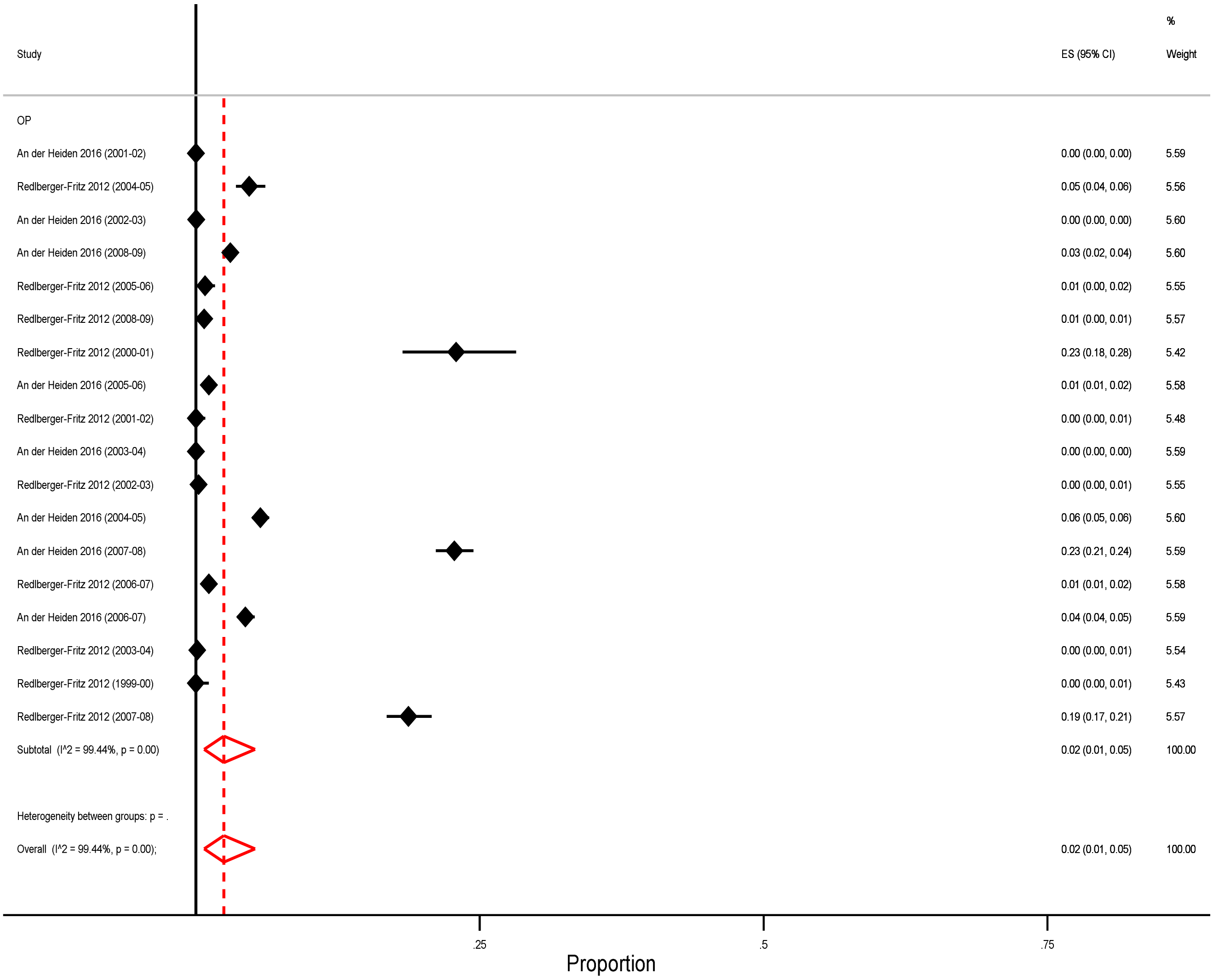

No studies of pre-pandemic H1N1 in in-patients.

S15b: Forest plot (studies from literature review): proportion of post-pandemic influenza A(H1N1) viruses subgroup analysis by healthcare setting in patients seeking care in Europe

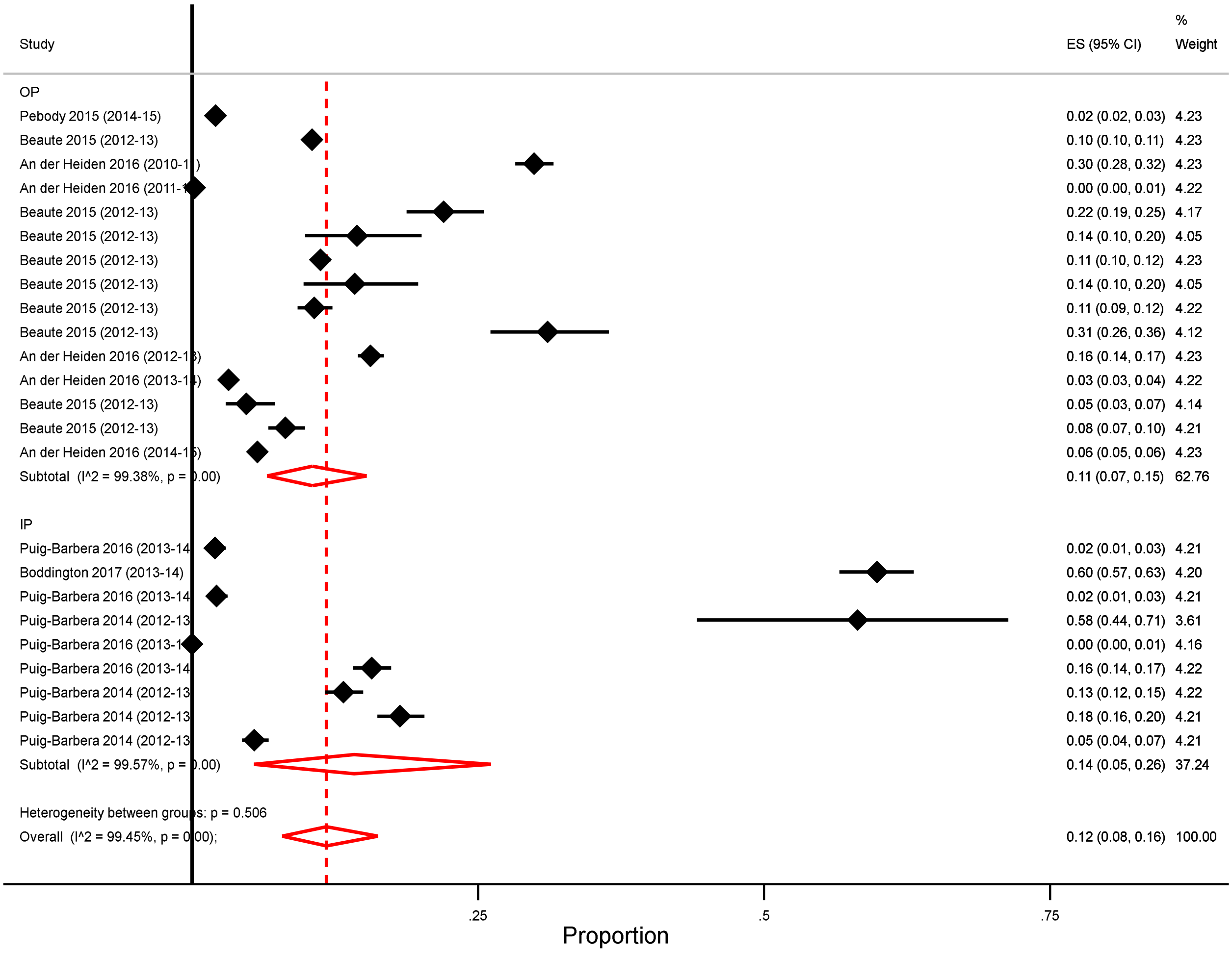

S16a: Forest plot (studies from literature review): proportion of pre-pandemic influenza A(H1N1) viruses by age groups in outpatients seeking care in Europe

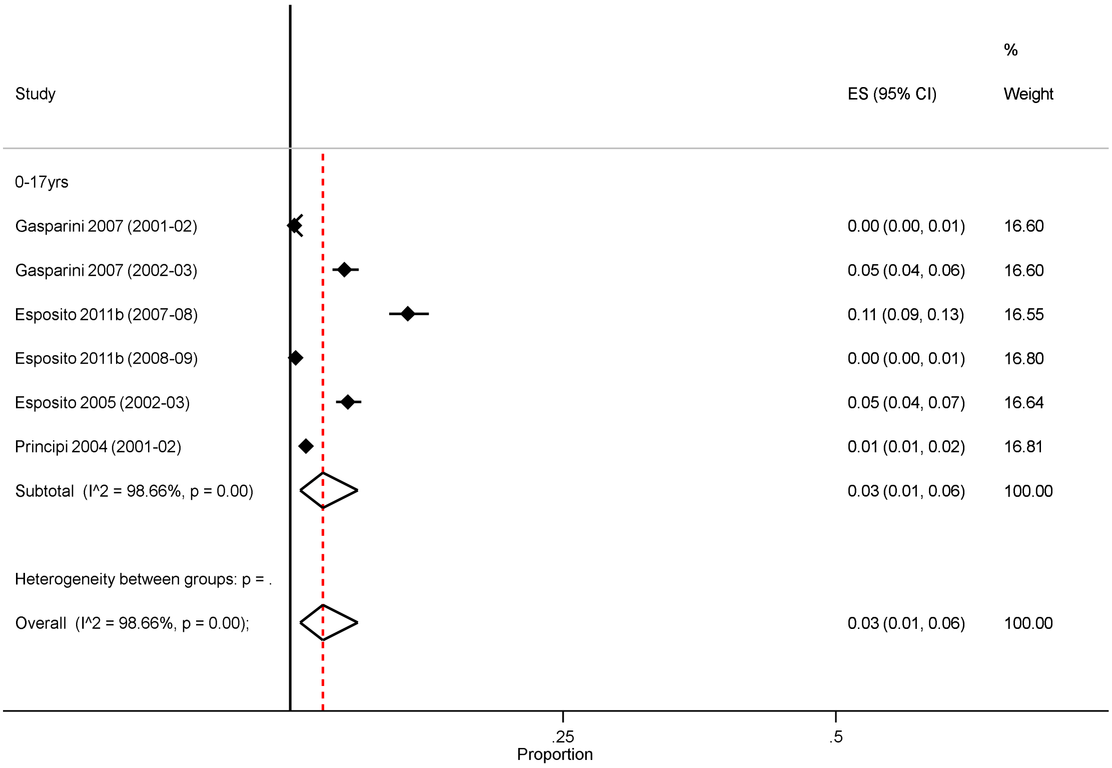

S16b: Forest plot (studies from literature review): proportion of post-pandemic influenza A(H1N1) viruses by age groups in out-patients seeking care in Europe

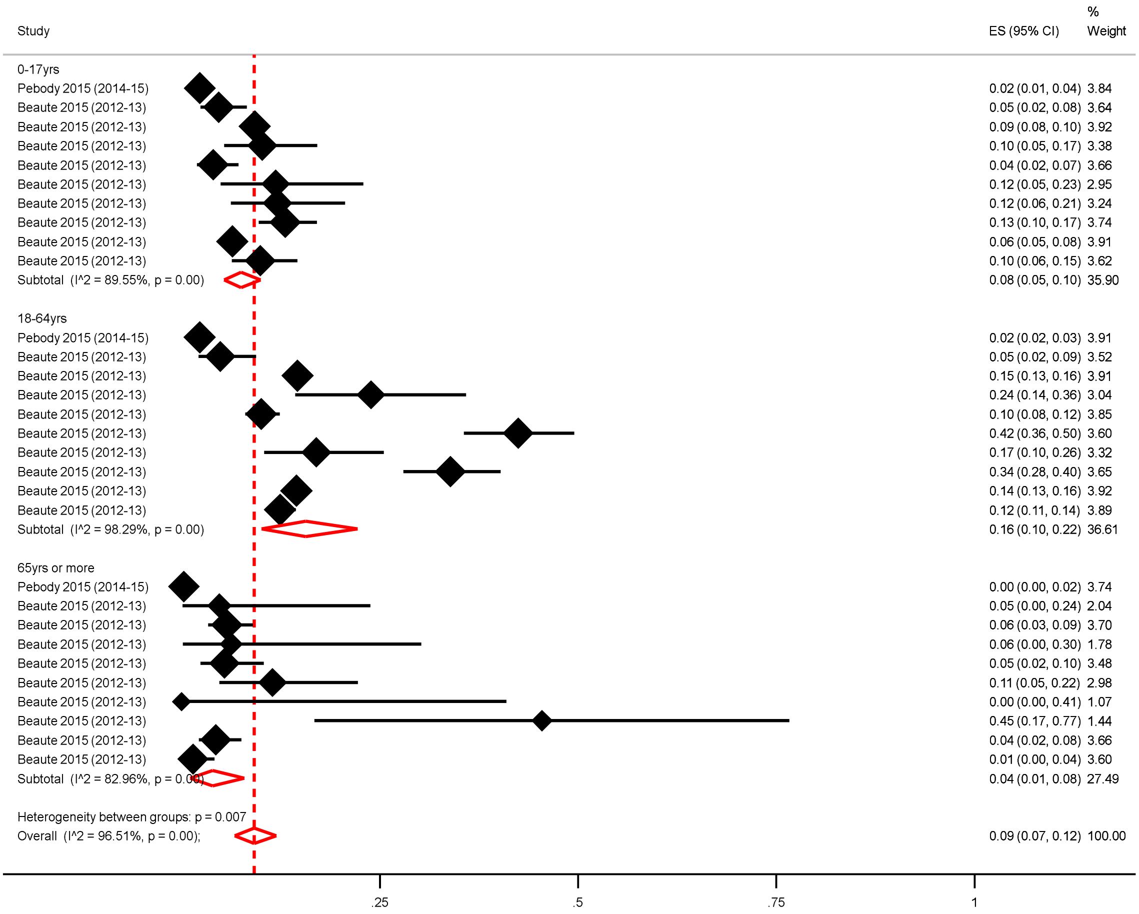

S17a: Forest plot (studies from literature review): proportion of pre-pandemic influenza A(H1N1) viruses by age groups in inpatients seeking care in Europe

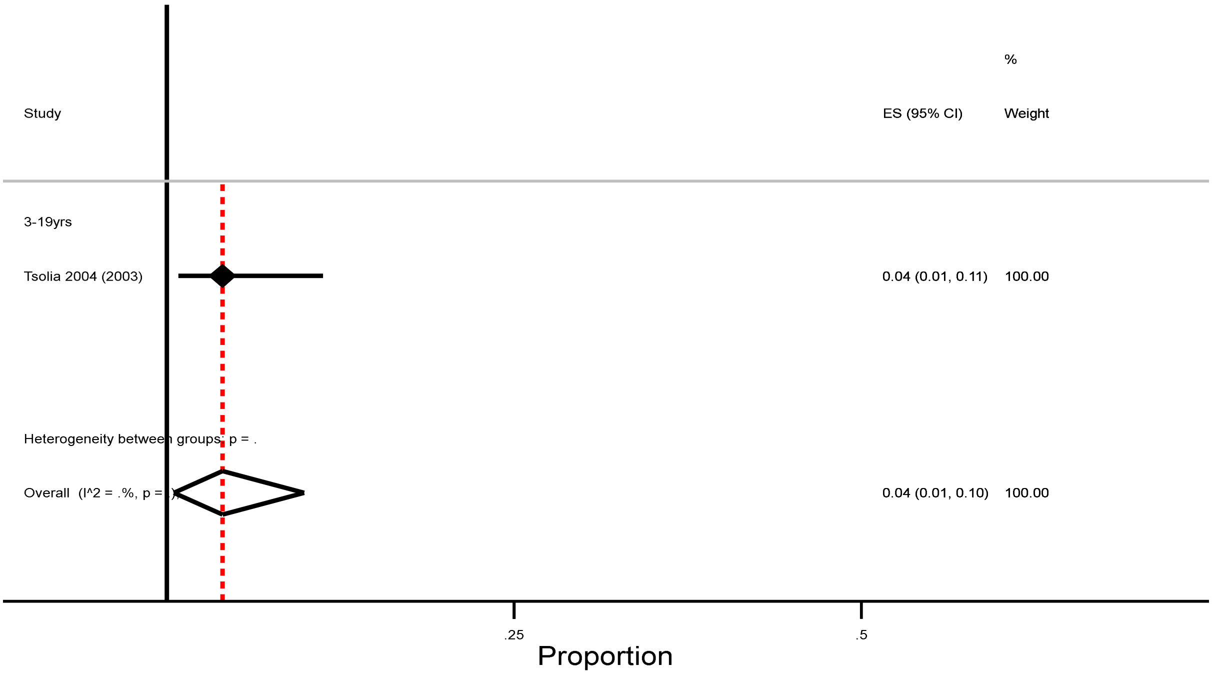

S17b: Forest plot (studies from literature review): proportion of post-pandemic influenza A(H1N1) viruses by age groups in inpatients seeking care in Europe

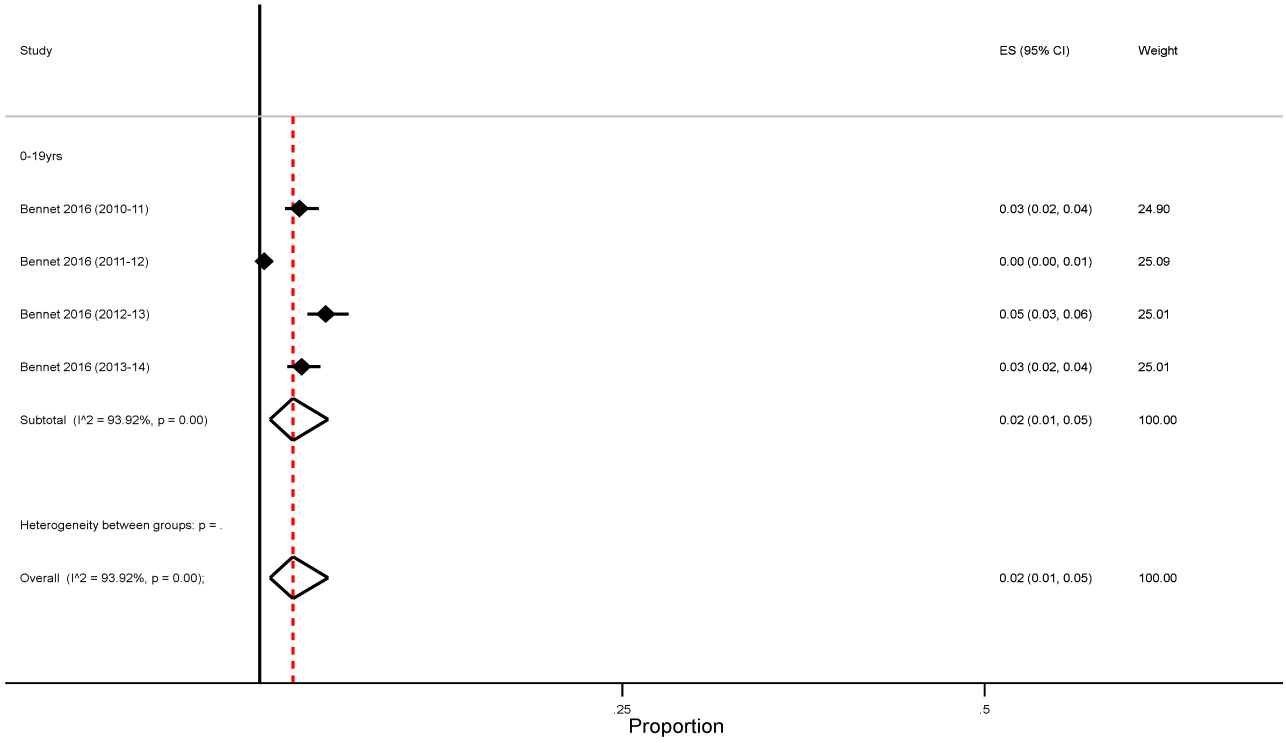

S18: Forest plot (studies from literature review): proportion of influenza A(H3N2) viruses subgroup analysis by healthcare setting in patients seeking care in Europe

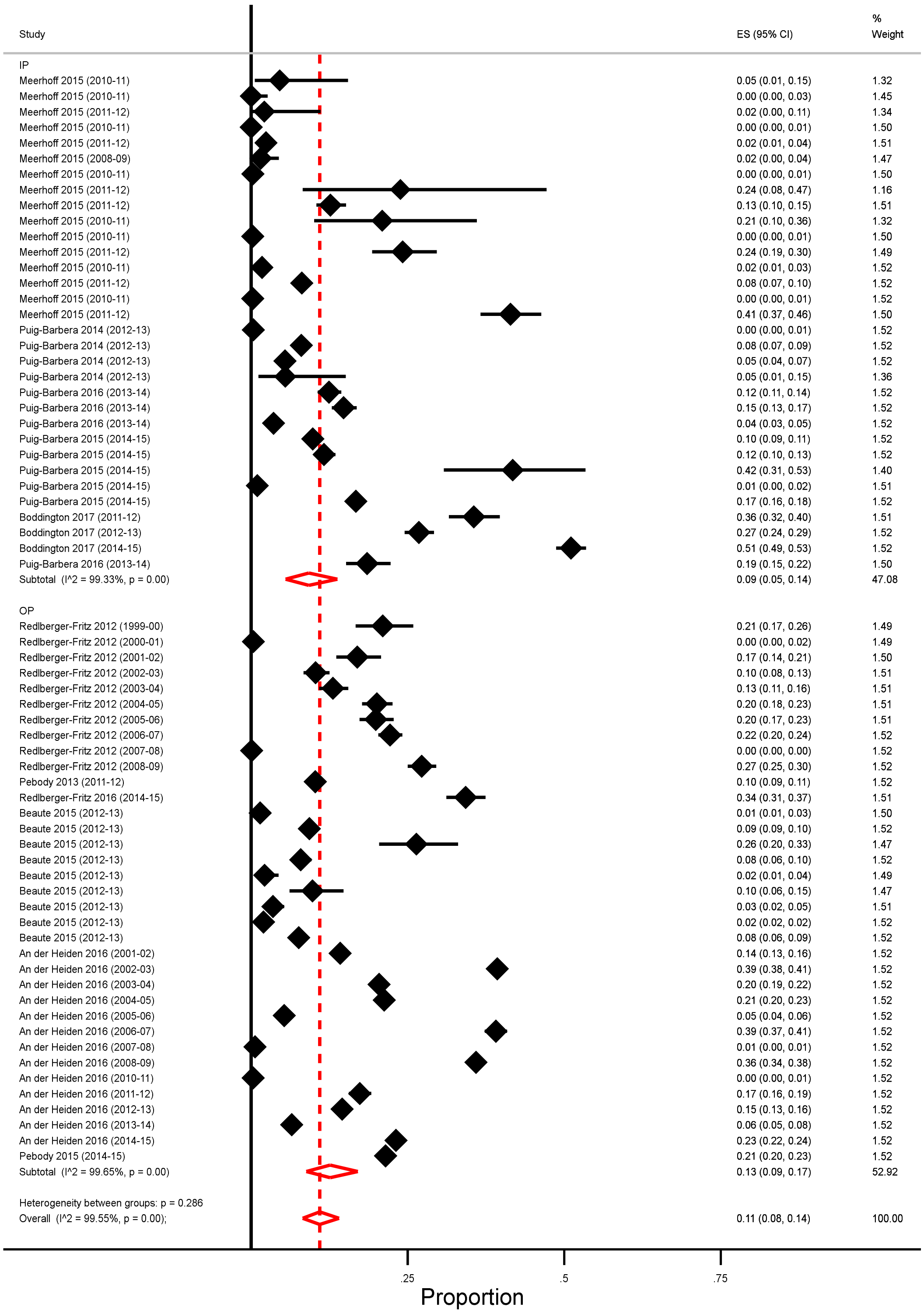

S19: Forest plot (studies from literature review): proportion of Influenza A(H3N2) viruses by age groups in Outpatients seeking care in Europe

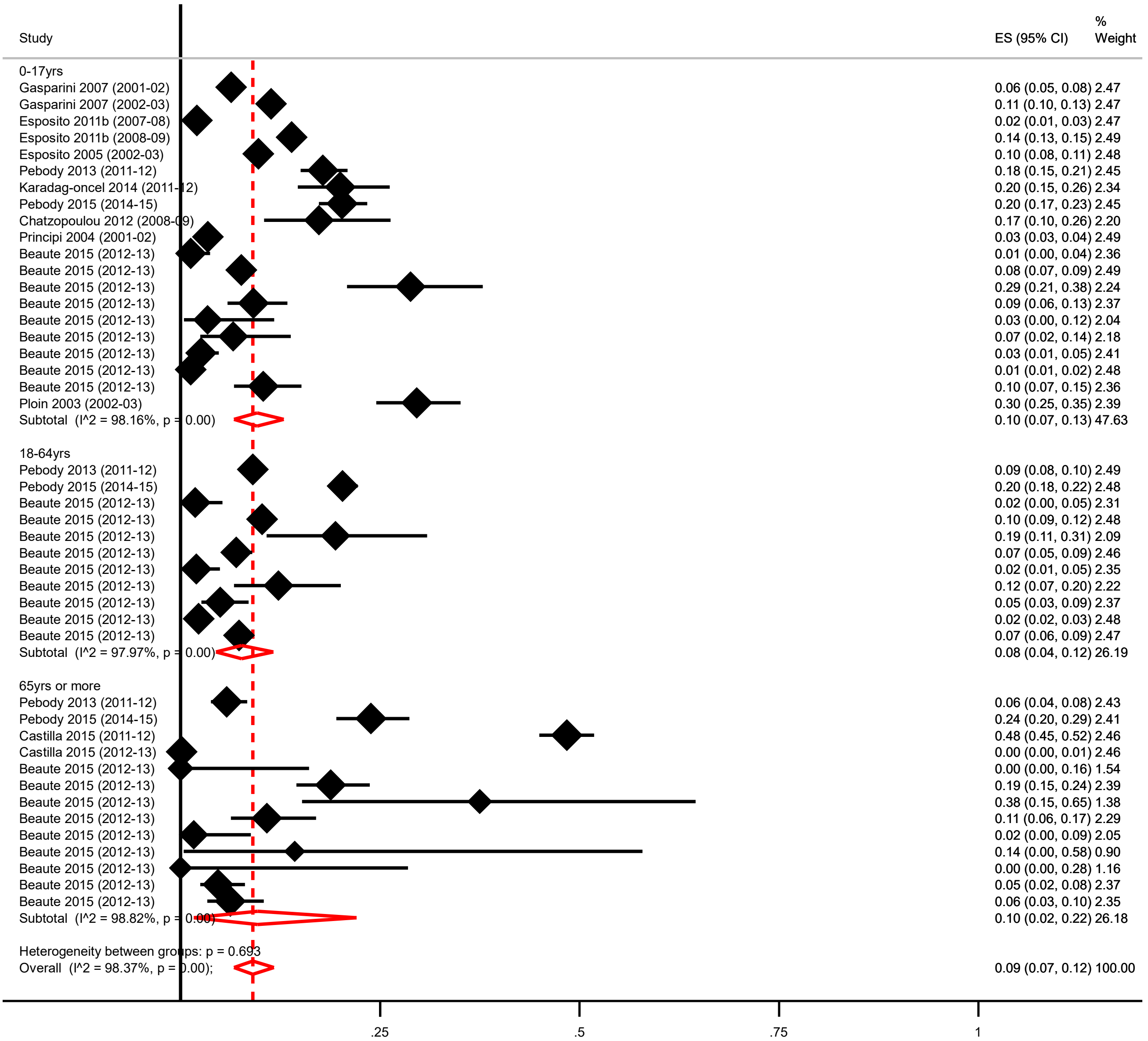

S20: Forest plot (studies from literature review): proportion of influenza A(H3N2) viruses by age groups in inpatients seeking care in Europe

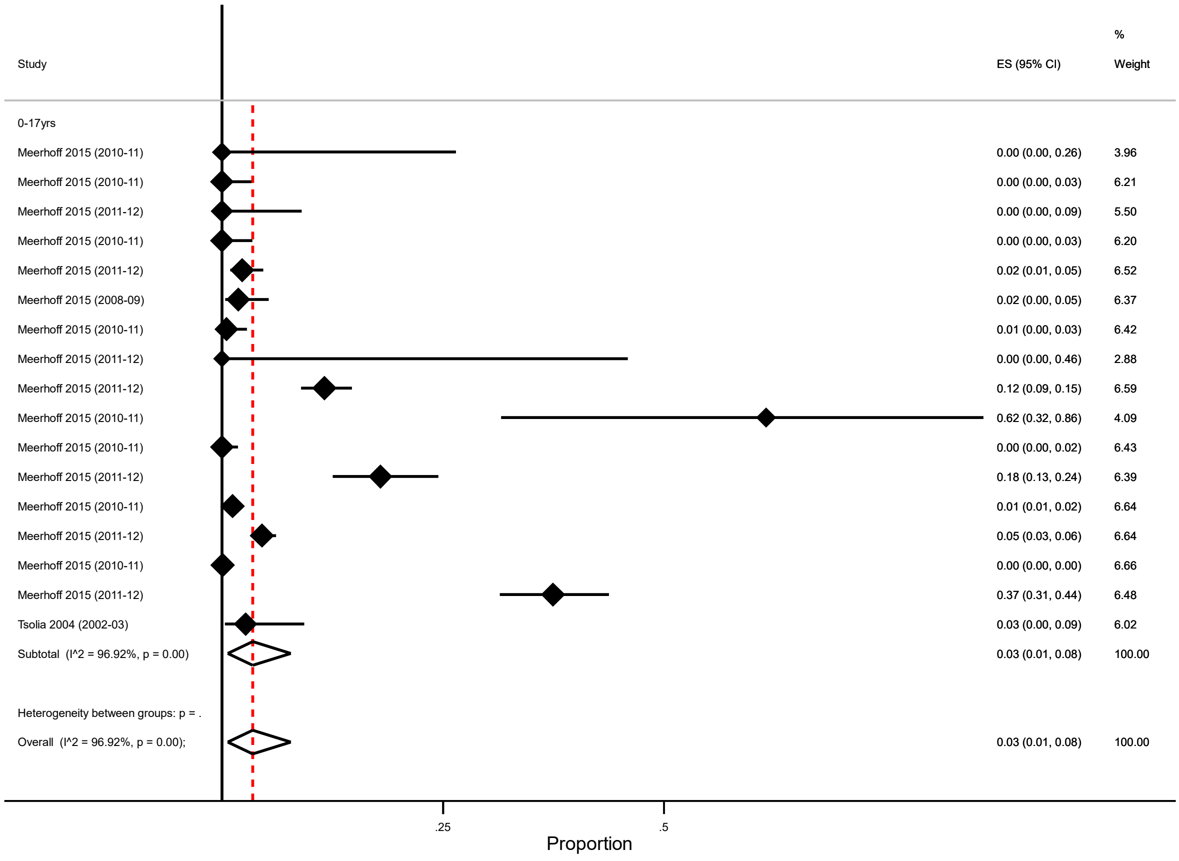

S21: Proportion of confirmed influenza by type/subtype for influenza seasons between 1999-2000 and 2014-2015 (literature review)

Season	Proportion laboratory-confirmed influenza % (95% CI)			
	Influenza A	Influenza A(H1N1)	Influenza A(H3N2)	Influenza B
1999-2000	36 (31-42)	0 (0-1)	21 (17-26)	0 (0-0)
2000-2001	38 (33-44)	23 (18-28)	0 (0-2)	4 (2-7)
2001-2002	15 (13-16)	0 (0-0)	15 (13-16)	15 (14-16)
2002-2003	42 (40-43)	0 (0-0)	34 (32-25)	6 (6-7)
2003-2004	31 (20-43)	0 (0-0)	19 (17-20)	0 (0-0)
2004-2005	22 (17-27)	5 (5-6)	21 (20-22)	4 (1-8)
2005-2006	11 (5-18)	1 (1-1)	9 (8-10)	14 (6-26)
2006-2007	29 (17-43)	3 (2-3)	32 (31-33)	0 (0-0)
2007-2008	20 (16-24)	21 (20-22)	0 (0-1)	10 (3-20)
2008-2009	30 (23-37)	2 (2-3)	19 (7-33)	6 (3-9)
2010-2011	24 (23-25)	30 (28-32)	1 (0-1)	14 (9-20)
2011-2012	47 (18-77)	0 (0-0)	16 (10-23)	1 (0-2)
2012-2013	23 (18-27)	14 (12-17)	7 (4-11)	17 (12-23)
2013-2014	16 (12-21)	9 (1-24)	10 (6-16)	2 (0-5)
2014-2015	27 (22-28)	4 (3-4)	21 (13-30)	9 (6-10)

S22: Proportion of confirmed influenza by type/subtype for influenza seasons between 2004-2005 and 2017-2018 (TESSy ILI/ARI data)

Season	Proportion laboratory-confirmed influenza % (95% CI)			
	Influenza A	Influenza A(H1N1)	Influenza A(H3N2)	Influenza B
2004-2005	24 (19-28)	2 (1-4)	15 (11-20)	7 (5-8)
2005-2006	8 (6-10)	1 (1-2)	3 (2-4)	15 (11-19)
2006-2007	30 (23-37)	1 (0-1)	18 (12-26)	1 (0-1)
2007-2008	20 (16-24)	13 (9-19)	0 (0-0)	10 (7-13)
2008-2009	26 (20-32)	1 (0-1)	15 (10-21)	6 (5-8)
2010-2011	21 (18-24)	16 (13-19)	1 (0-1)	13 (11-16)
2011-2012	23 (18-28)	0 (0-1)	19 (15-23)	3 (2-4)
2012-2013	20 (17-23)	10 (8-12)	5 (4-7)	17 (13-21)
2013-2014	21 (16-26)	8 (5-11)	10 (7-13)	1 (0-1)
2014-2015	23 (19-28)	4 (2-5)	15 (12-19)	11 (9-13)
2015-2016	22 (19-25)	17 (14-20)	1 (1-2)	12 (9-16)
2016-2017	32 (28-37)	0 (0-0)	27 (22-32)	3 (2-4)
2017-2018	12 (10-14)	6 (4-7)	3 (2-4)	25 (21-29)
